# Horizontal acquisition of multiple mitochondrial genes from a parasitic plant followed by gene conversion with host mitochondrial genes

**DOI:** 10.1186/1741-7007-8-150

**Published:** 2010-12-22

**Authors:** Jeffrey P Mower, Saša Stefanović, Weilong Hao, Julie S Gummow, Kanika Jain, Dana Ahmed, Jeffrey D Palmer

**Affiliations:** 1 Department of Biology, Indiana University Bloomington, Bloomington, Indiana 47403, USA; 2 Center for Plant Science Innovation, University of Nebraska-Lincoln, Lincoln, Nebraska 68588, USA; 3 Department of Biology, University of Toronto Mississauga, Mississauga, Ontario L5L 1C6, Canada; 4 Department of Laboratory Medicine and Pathobiology, University of Toronto, Toronto, Ontario, M5G 1L5, Canada

## Abstract

**Background:**

Horizontal gene transfer (HGT) is relatively common in plant mitochondrial genomes but the mechanisms, extent and consequences of transfer remain largely unknown. Previous results indicate that parasitic plants are often involved as either transfer donors or recipients, suggesting that direct contact between parasite and host facilitates genetic transfer among plants.

**Results:**

In order to uncover the mechanistic details of plant-to-plant HGT, the extent and evolutionary fate of transfer was investigated between two groups: the parasitic genus *Cuscuta *and a small clade of *Plantago *species. A broad polymerase chain reaction (PCR) survey of mitochondrial genes revealed that at least three genes (*atp1*, *atp6 *and *matR*) were recently transferred from *Cuscuta *to *Plantago*. Quantitative PCR assays show that these three genes have a mitochondrial location in the one species line of *Plantago *examined. Patterns of sequence evolution suggest that these foreign genes degraded into pseudogenes shortly after transfer and reverse transcription (RT)-PCR analyses demonstrate that none are detectably transcribed. Three cases of gene conversion were detected between native and foreign copies of the *atp1 *gene. The identical phylogenetic distribution of the three foreign genes within *Plantago *and the retention of cytidines at ancestral positions of RNA editing indicate that these genes were probably acquired via a single, DNA-mediated transfer event. However, samplings of multiple individuals from two of the three species in the recipient *Plantago *clade revealed complex and perplexing phylogenetic discrepancies and patterns of sequence divergence for all three of the foreign genes.

**Conclusions:**

This study reports the best evidence to date that multiple mitochondrial genes can be transferred via a single HGT event and that transfer occurred via a strictly DNA-level intermediate. The discovery of gene conversion between co-resident foreign and native mitochondrial copies suggests that transferred genes may be evolutionarily important in generating mitochondrial genetic diversity. Finally, the complex relationships within each lineage of transferred genes imply a surprisingly complicated history of these genes in *Plantago *subsequent to their acquisition via HGT and this history probably involves some combination of additional transfers (including intracellular transfer), gene duplication, differential loss and mutation-rate variation. Unravelling this history will probably require sequencing multiple mitochondrial and nuclear genomes from *Plantago*.

See Commentary: http://www.biomedcentral.com/1741-7007/8/147.

## Background

Horizontal gene transfer (HGT) is the transmission of genes across species boundaries and/or mating barriers. HGT plays a major role in prokaryotic evolution, where it occurs through such well-studied processes as transformation, conjugation and transduction [1,2]. HGT is also relatively common and evolutionarily important in certain phagotrophic protists [3-6], with food prey often serving as the source of these transferred genes [7]. However, relatively few cases of HGT have been reported in most multicellular, non-phagotrophic eukaryotes and little is known about the mechanisms of transfer [8].

For the most part, HGT in plants is comparable to that of other multicellular eukaryotes - it is a rare phenomenon. Despite intense investigations of genetically modified crops, due to the potential for transgene escape, there are very few examples of plants donating genes to any non-plant species [9]. Other than the massive migration of bacterial genes into the nucleus after the endosymbiotic establishment of the mitochondrion and plastid [10-12], the transfer of non-plant genes into plants is also uncommon. Perhaps the best examples come from the transfer of infectious plasmids from *Agrobacterium *[13,14], the transfer of a mobile group I intron from a fungus [15,16] and the ancient transfer of a few fungal genes into angiosperm nuclear genomes [17]. Horizontal transmission between plants, at the nuclear level, has so far been documented for only a few transposable elements and genes [18-21]. At the plastid level, plant-to-plant HGT is apparently non-existent or at least exceedingly rare. No cases were discovered after the examination of 42 complete plastid genomes from representative green plants and red algae plus a single glaucophyte [22] and no reports have emerged from the many subsequently-sequenced plastid genomes.

Although plastid and nuclear gene transfer appears to be rare among plants, a significant body of evidence indicates that plant-to-plant transfer of mitochondrial genes occurs with surprising frequency (for examples see [23-31]). In most cases, the mechanisms of mitochondrial transfer remain speculative, with possibilities including: direct contact between donor and recipient plants; uptake of DNA from the environment; and transfer of DNA via vectors such as viruses, bacteria or fungi [23-25]. However, various lines of evidence suggest that mitochondrial HGT is facilitated by direct cell-to-cell contact between different species, involving parasitism and, perhaps, grafting [32]. Several studies have suggested, largely on phylogenetic grounds, that plant mitochondrial genes move from host to parasite [26,28] or from parasite to host [27,29]. It has been speculated that haustorial connections, which allow the passage of macromolecules, viruses and phytoplasmas between parasitic plants and their host plants, may also facilitate HGT [26-28]. Evidence that experimental grafting enables frequent plastid gene transfer suggests that it may also be an evolutionarily important route of mitochondrial HGT [33].

Despite these important findings, much about the mechanism of mitochondrial horizontal transfer remain largely unclear. One reason is that it is not known whether the transferred genetic material is DNA or RNA. In a related phenomenon - intracellular gene transfer - it has been established that mitochondrial genes can integrate into the nucleus through either an RNA intermediate [34,35] or directly via DNA [36-38]. Therefore, either or both routes may also be available for HGT. Another largely unanswered question is whether the nucleic acid is nakedly transferred or packaged inside a vector. Double-stranded genomic DNA is known to persist for thousands of years in specific environmental conditions [39,40], whereas single-stranded RNA or complementary DNA (cDNA) is not expected to fare as well. Potential vectors for a packaged transfer include viruses, bacteria, fungi, insects and mitochondria themselves. This last route is supported by two observations: transfer may occur by direct contact between donor and recipient plants; and plant mitochondria (but not plastids) are well known to fuse [41,42], accompanied by intergenomic recombination in somatic hybrids [43,44]. Finally, which of the three plant genomes is the site of integration of foreign plant mitochondrial genes is largely unexplored. Although some analyses have provided evidence for mitochondrial integration [23,25,30,31], the possibility that foreign sequences of mitochondrial origin reside, instead, in the nucleus has been raised [45,46] because nuclear (but not plastid) genomes readily incorporate sequences of mitochondrial origin, at least via intracellular gene transfer [36-38].

We previously reported on two independent cases of horizontal transfer of the mitochondrial *atp1 *gene from different parasitic plant groups into genus *Plantago *[27]. In one case, we identified the donor group as the parasitic genus *Cuscuta *(dodders; Convolvulaceae) and the recipient as the common ancestor of a small clade of three closely-related *Plantago *species, *Plantago coronopus, P. macrorhiza *and *P. subspathulata *(out of 43 species sampled). As this transfer event was recent, and the donor and recipient lineages are well-defined, it is an excellent case in which to address some of the outstanding mechanistic issues of HGT. Furthermore, we should be able to distinguish between DNA and RNA mediated mechanisms of transfer by examining historical patterns of cytidine to uridine (C-to-U) RNA editing which occurs in almost all plant mitochondrial transcripts [47-53]. In the course of this investigation we discovered two additional mitochondrial genes that have been transferred from *Cuscuta *into the same group of three closely-related *Plantago *species, which suggests that a large portion of the mitochondrial genome was transferred. Phylogenetic and other analyses shed light on the mechanism of transfer and also reveal an intriguingly complex history of these genes subsequent to their acquisition.

## Results

### Horizontal transfer of multiple genes from *Cuscuta *to *Plantago*

In order to determine whether other mitochondrial genes were transferred between *Cuscuta *and *Plantago*, potentially via the same transfer event, a polymerase chain reaction (PCR)-based survey of 38 protein and ribosomal RNA (rRNA) genes that were present in the mitochondrial genome of the ancestral eudicot [54] was undertaken for *P. coronopus *and *C. gronovii *using a comprehensive set of mitochondrial primers that had been developed earlier [25]. We succeeded in amplifying (and sequencing) only 10 of the 38 genes from both species. These genes were subjected to preliminary phylogenetic analyses using sequence data available in GenBank (data not shown). In addition to the previously identified *atp1 *gene, the *atp6 *and *matR *genes also showed clear evidence of horizontal transfer from *Cuscuta *to *Plantago *in these preliminary analyses, whereas the other seven genes are evidently native to *Plantago*. We failed to recover a PCR product for the remaining 28 genes in the survey from *P. coronopus *and these were not evaluated further. The high rate of amplification failure in this species is probably due to the poor hybridization of primers to the exceptionally divergent mitochondrial genes in *Plantago *[55-58] and to gene loss from the mitochondrial genomes, which is a common phenomenon for many plant species [11,54]. The high level of point mutations and indels in the putative horizontally transferred genes identified here (Additional File 1) suggests that additional HGT candidates may also have been missed as a result of poor primer hybridization.

**Figure 1:**
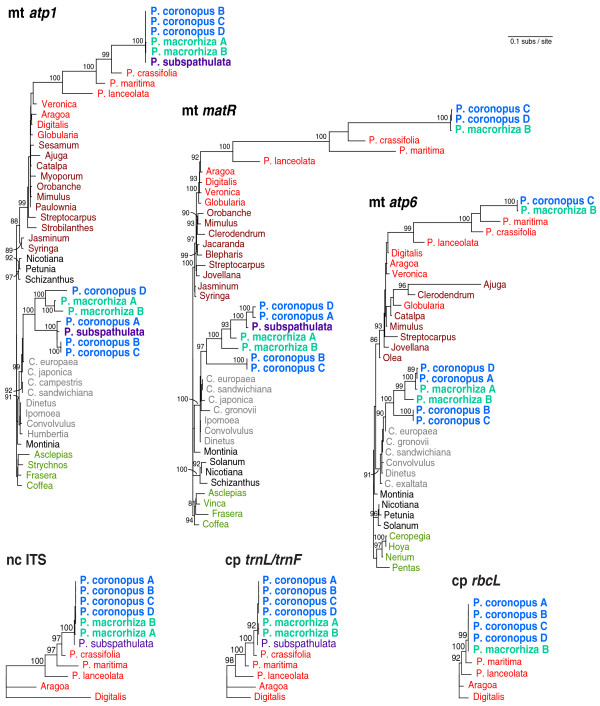
**Multi-gene horizontal transfer from *Cuscuta *to *Plantago***. Maximum likelihood (ML) phylogenetic analyses of three mitochondrial genes [*atp1 *(1272 bp), *atp6 *(615 bp) and *matR *(1878 bp)], two plastid regions [*rbcL *(984 bp) and *trnL*/*trnF *intergenic spacer (367 bp)] and the nuclear internal transcribed spacer [ITS (696 bp)] are shown. All trees are drawn to the same scale (scale bar is at top right). Bootstrap values >80% from 100 ML replicates are shown. 'P' denotes *Plantago *and 'C' *Cuscuta*. Lamiales are coloured in shades of red, with lighter red for Plantaginaceae and darker red for the remaining families, except for three *Plantago *species involved in horizontal gene transfer, which are in blue (*Plantago coronopus*), turquoise (*P. macrorhiza*) or violet (*P. subspathulata*). Solanales are in shades of grey, with lighter grey for Convolvulaceae and darker grey for the remaining families. Gentianales are in green and served as outgroups to root the mitochondrial trees.

For all three genes for which preliminary analyses indicated HGT, homologs from diverse representatives of the Lamiales (including *Plantago *and other Plantaginaceae), Solanales (including *Cuscuta *and other Convolvulaceae) and Gentianales were PCR amplified and sequenced or collected directly from GenBank (Additional File 2). In addition, in order to evaluate the within-species diversity of HGT, these genes were sequenced from up to four different *P. coronopus *lines (referred to for convenience as A, B, C and D) and from two different *P. macrorhiza *lines (A and B; Additional File 3). These sequences were aligned and phylogenetic analyses were performed using maximum likelihood (Figure 1). In each case, a *Plantago *clade of species was found nested within Lamiales (with strong bootstrap support of 93%-100%) and, more specifically, within Plantaginaceae (with strong support for *matR *only), the expected position for native *Plantago *genes. For *atp1*, native copies were previously isolated from 43 species of *Plantago *[27] but only six species are shown here: the focal species in the '*P. coronopus *clade' (*P. coronopus*, *P. macrorhiza *and *P. subspathulata*) and three additional species (*P. crassifolia, P. maritima *and *P. lanceolata*) that represent lineages of increasing divergence relative to the focal group [59,60]. For *atp6 *and *matR*, sampling was limited to the species shown: no additional species were attempted or sequenced.

In addition to the native copies of *atp1, atp6*, and *matR*, a second copy of each of these genes was amplified from *P. coronopus *and *P. macrorhiza*. A second copy of *atp1 *and *matR *was also amplified from *P. subspathulata*. These additional copies all group in an anomalous position, together with or within Convolvulaceae (Solanales), with high bootstrap support (Figure 1). Moreover, in each case, they are nested within the parasitic genus *Cuscuta*, a position that is weakly supported for two genes and strongly supported (90%) for *atp6 *(Figure 1). Taken together, the strongly supported placement of these sequences within Convolulaceae, their close affinity with *Cuscuta *and their strongly supported exclusion from the *Plantago *clades of native homologs indicate that all three genes were probably transferred horizontally from a species of *Cuscuta *into the common ancestor of *P. coronopus*, *P. macrorhiza *and *P. subspathulata*. [Note that none of the three genes was recovered from either *P. crassifolia *or *P. maritima*, the two successively sister lineages of the *P. coronopus *clade.]

Errors in phylogenetic reconstruction can lead to erroneous claims of HGT. However, this is unlikely to be the case here. For each gene, the phylogenetic position of the putative foreign copies is robust; their placement was unaffected by choice of phylogenetic method (maximum likelihood, parsimony or neighbour-joining), by elimination of predicted sites of RNA editing from the data, by removing the native *Plantago *genes from the data or by broadening the taxon sampling to representatives from across core eudicots (Additional File 4). Furthermore, for all three genes, the Shimodaira-Hasegawa test [61], which is a likelihood-based statistical test of alternative tree topologies, strongly rejected (*P *< 0.05) the placement of the foreign clade within Plantaginaceae in the position expected if the clade arose by gene duplication rather than by HGT (Additional File 5). More generally, with the exception of the anomalous placement of the foreign clade of *Plantago *sequences, the strongly supported relationships throughout the rest of the three gene trees are consistent with the currently accepted organismal phylogeny [62], arguing against any pervasive phylogenetic issues.

It has been argued that nuclear-encoded fragments of mitochondrial DNA (numts) may sometimes be mistaken for horizontally transferred DNA [45,46], perhaps due to phylogenetic artifacts arising from the very different mutation rates in the two genomes. In general, however, numts should behave like gene duplications in phylogenetic analyses and group with their mitochondrial progenitor sequences. As described above, this is clearly not the case for the *Plantago *sequences. Indeed, the strongly supported placement of the putatively foreign *Plantago *copy of all three mitochondrial genes with Convolvulaceae (Solanales), convincingly apart from the native *Plantago *homologs - which are well embedded within multiple strongly supported lineages of Lamiales (Figure 1) - makes the numt phylogenetic artifact hypothesis untenable in the case of *Plantago*.

Misidentification or contamination of DNA is another problem that can lead to incorrect inferences of HGT. However, wholesale misidentification can be clearly ruled out by the fact that intact sequences grouping within Plantaginaceae have been obtained for multiple loci (including 10 mitochondrial, six plastid and two nuclear loci from *P. coronopus*) from all *Plantago *samples used in this study (Additional File 3) and during our previous investigations of substitution rates [56]. Contamination of DNA stocks with some unknown secondary source can also be discounted. All horizontally acquired copies were amplified and sequenced at least twice from each DNA sample. Furthermore, at least two independent DNA samples were prepared from each plant grown at the University of Nebraska-Lincoln (Additional File 3). Finally, of all sequenced loci, only *atp1, atp6 *and *matR *gave any indication that more than one gene copy was present, and all of these additional copies are clearly pseudogenes (see the next section). By contrast, contamination with DNA from a different plant would be expected to produce multiple gene copies from more than just three of the 38 surveyed mitochondrial genes, and all of the additional copies should look functional.

### Horizontally transferred genes are unexpressed pseudogenes

**Figure 2:**
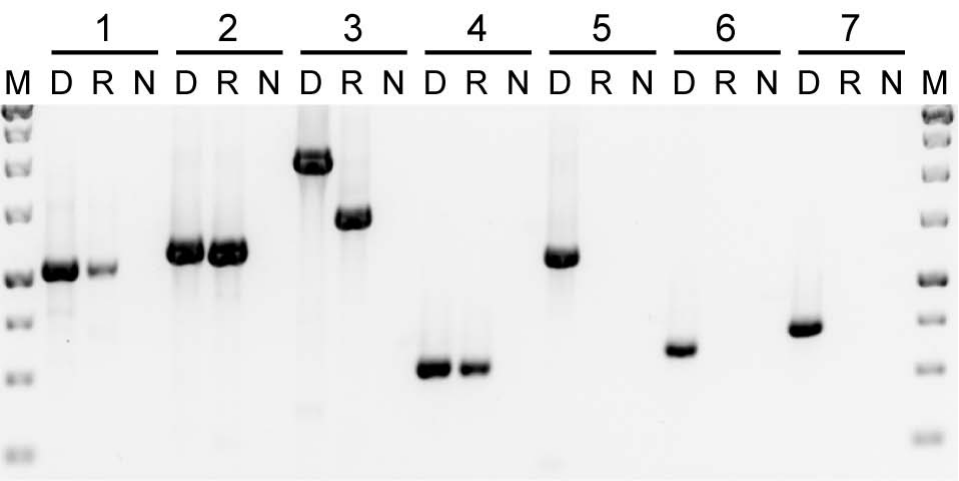
**Expression of native and foreign genes**. Primers were used to amplify the following genes from *Plantago coronopus *line C: (1) plastid *rbcL; *(2) native *atp1; *(3) native *cox1; *(4) native *matR; *(5) foreign *atp1; *(6) foreign *atp6; *and (7) foreign *matR*. Three templates were used for amplification: DNA (D), reverse-transcribed RNA (R) and a negative control (N).

An unusual characteristic of *Plantago *mitochondrial genes is that their nucleotide sequences are highly divergent due to the unusually high mutation rates [55-58]. This is evident here for the native copies of *atp1*, *atp6 *and *matR *(Figure 1). Despite this divergence, all of the native *Plantago *sequences are intact with no frameshifting indels or internal stop codons (Additional File 1). In addition, all four native mitochondrial and plastid genes from *P. coronopus *line C examined by reverse transcription (RT)-PCR - including the native *atp1 *and *matR *genes - were found to be transcribed (Figure 2), as were the native *atp1 *and *matR *genes from *P. macrorhiza *line B (data not shown). The smaller size and the presence of three C-to-U changes in the *P. coronopus cox1 *cDNA relative to genomic DNA (Figure 2, lane 3) indicate that the transcript had undergone intron splicing and RNA editing, which verifies that the cDNA sample was indeed derived from RNA. Thus, the native *atp1*, *atp6 *and *matR *genes are likely to be *bona fide *genes encoding functional *Plantago *mitochondrial proteins.

All of the foreign genes are also divergent relative to other taxa (Figure 1). However, they are almost certainly nonfunctional because they contain indels that disrupt the reading frame and often introduce premature stop codons (Additional File 1). Despite the clear indications of pseudogenization at the DNA level, it is possible that the horizontal copies are nonetheless still transcribed. We assayed all three foreign genes from *P. coronopus *line C and *P. macrorhiza *line B and could not detect transcripts from any of them (Figure 2 and data not shown). Thus, it appears that the horizontally transferred genes are unexpressed pseudogenes.

### Phylogenetic incongruence within clades of horizontally acquired genes

We amplified and sequenced native copies of three mitochondrial genes (*atp1, atp6 *and *matR*), two plastid regions (*rbcL *and intergenic *trnL/trnF*) and the internal transcribed spacer (ITS) of the nuclear ribosomal repeat from 4-6 species of *Plantago*, including multiple *P. coronopus *and *P. macrorhiza *lines for most genes, and at least two other members of Plantaginaceae (Figure 1). Phylogenetic relationships for all native loci are consistent with previous phylogenies [59,60,63], although it should be noted that the particular relationship among *P. coronopus, P. macrorhiza *and *P. subspathulata *is not resolved here or elsewhere ([59,60]; JPM and JDP, unpublished data).

We also sequenced foreign copies of all three mitochondrial genes from the six different *P. coronopus *and *P. macrorhiza *lines. Surprisingly, the sequences from the different *P. coronopus *lines were never monophyletic and the *P. macrorhiza *lines were not monophyletic for the *atp6 *and *matR *genes (Figure 1). Furthermore, the phylogenetic relationship among the foreign *atp1 *sequences varies dramatically compared to the topology for *matR *and *atp6*. For example, *P. coronopus *line A groups with lines B and C for *atp1 *but with line D for *atp6 *and *matR*. The inconsistent and reproducible phylogenetic pattern for various lines rules out seed misidentification as the source of taxonomic conflict and indicates a complicated evolutionary history for these pseudogenes.

### Complex patterns of sequence divergence in horizontally transferred genes

An unexpectedly complex history of the foreign genes following their arrival in *Plantago *is also indicated by patterns of sequence divergence (Figure 1, Additional File 1). There are very few if any nucleotide differences among the native mitochondrial copies of *atp1, atp6 *and *matR *within the *P. coronopus *clade of three species. In fact, the most divergent sequences within the *P. coronopus *clade for native mitochondrial genes are >99% identical to one another, which is comparable to divergence levels of plastid sequences sampled from the same plants. In stark contrast, the foreign genes are much more divergent. The most divergent sequences for the foreign genes are only 90%, 92% and 84% identical for *atp1*, *atp6 *and *matR*, respectively, and these extremes of divergence actually reside among the multiple lines of *P. coronopus *sampled.

In order to explore this rate variation further, we used a codon-based model of sequence evolution to evaluate synonymous (dS) and non-synonymous (dN) divergence for native and foreign homologs from particular species pairs (Table 1). These analyses corroborate patterns observed by simple comparison of sequence identities: the native mitochondrial genes are essentially identical to one another, whereas the foreign homologs are much more divergent. Levels of synonymous divergence are 15-25 times higher between the foreign pseudogenes from *P. coronopus *and *P. macrorhiza *compared to native genes from the same species. Results from individual gene dS analyses are generally consistent with the combined analysis (Table 1). Non-synonymous values are more variable, which is probably the result of differential selection pressures acting on the genes, even the pseudogenes to some extent because ω < 1 in all cases.

**Table 1:** Pairwise divergence estimates for native and foreign homologs in *Plantago*.

Gene	dN ± SE	dS ± SE	*ω*
	(subs/100 sites)	(subs/100 sites)	
*Plantago coronopus *C versus *P. macrorhiza *B (native genes)			
*atp1*	0.15 ± 0.15	0.48 ± 0.47	0.32
*atp6*	0.00 ± 0.00	0.71 ± 0.68	0.00
*matR*	0.36 ± 0.18	0.59 ± 0.38	0.62
Combined	0.23 ± 0.10	0.58 ± 0.28	0.39
			
*P. coronopus *D versus *P. macrorhiza *B (foreign genes)			
*atp1*	5.4 ± 0.8	7.5 ± 1.7	0.72
*atp6*	3.9 ± 1.0	6.3 ± 2.2	0.62
*matR*	10.5 ± 1.5	14.1 ± 3.0	0.74
Combined	6.5 ± 0.7	9.2 ± 1.3	0.71
			
*P. coronopus *C versus *P. macrorhiza *B (foreign genes)			
*atp1*	7.5 ± 0.9	9.8 ± 2.1	0.77
*atp6*	5.5 ± 1.1	12.4 ± 3.5	0.44
*matR*	11.2 ± 1.5	23.5 ± 4.3	0.48
Combined	8.0 ± 0.7	14.3 ± 1.6	0.56

SE = standard error.

The differences in the substitution rate between the native and foreign homologs suggest that either: (1) they do not reside in the same genome; (2) they are in the same genome but there is intra-genomic variability in the rate of synonymous substitution; or (3) not all of the foreign copies isolated for a particular gene are orthologous to one another. However, it should be pointed out that certain subclades of foreign genes have levels of sequence divergence similar to native copies and relationships that are, in fact, consistent with organismal phylogeny. These are the subclades comprising *P. coronopus *B and C and *P. subspathulata *for *atp1 *and *P. coronopus *A and D and *P. macrorhiza *A for *atp6*.

### Multiple gene conversions of foreign *atp1 *by native *atp1*

**Figure 3:**
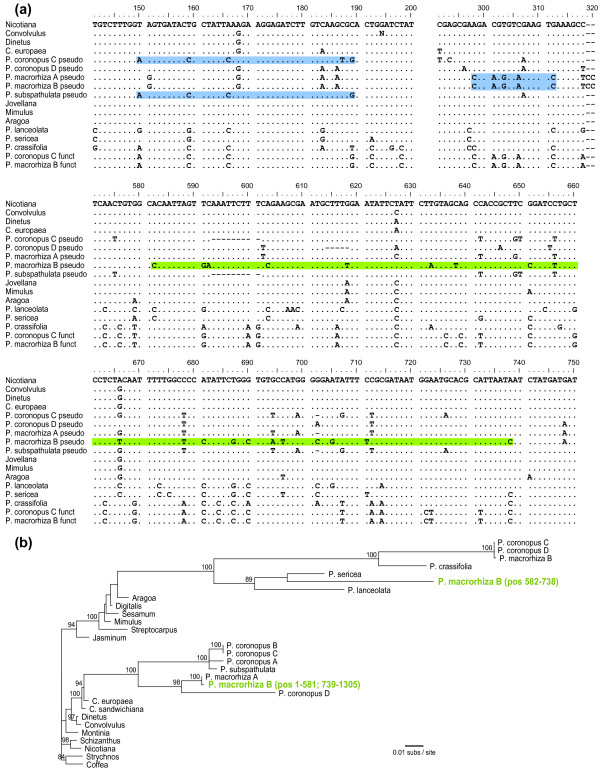
**Gene conversion between the native and foreign copies of *atp1***. (A) Sections of the *atp1 *alignment that demonstrate three examples of gene conversion of the foreign pseudogene copies by functional native copies from *Plantago*. Regions of putative gene conversion are highlighted in blue when the converting and converted sequences are from the same individual and in green when the interacting sequences are from apparently different species (see text). The full *atp1 *alignment is available in Additional File 1a. (B) Maximum likelihood (ML) phylogenetic analysis of the mosaic evolutionary history of the foreign *atp1 *copy from *Plantago macrorhiza *line B (green). For this analysis, the sequence spanning nt 582-738 was separated from the rest of the *P. macrorhiza *B foreign *atp1 *sequence and both regions were included in the data set as independent sequences. All other converted regions, including the two other regions shown in (A) and the region converted by plastid *atpA *(Additional File 1a; [71]), were excluded prior to phylogenetic analysis. The final alignment contained 1185 bp. Bootstrap values >80% from 100 ML replicates are shown.

The presence, owing to HGT, of two different copies of each of these three genes in the same plant prompted us to search systematically for evidence of any history of gene conversion between xenologs. The OrgConv program [64] failed to find evidence of gene conversion in *atp6 *and *matR *but detected three significant (*P *< 0.001) candidate cases of gene conversion in *atp1 *(Figure 3). The first conversion event was identified in the foreign genes from *P. coronopus *lines A-C and *P. subspathulata*. With the exception of a single autapomorphy in *P. coronopus *B and C, all four foreign genes are identical over a 40 nt region (positions 150-189) to the native *atp1 *genes of *P. coronopus *and *P. macrorhiza *but differ from the *Cuscuta *sequence (representing the donor group) at six or seven sites (Figure 3a). We therefore infer a native-to-foreign gene conversion event in the common ancestor of these four foreign *atp1 *sequences. The second example of gene conversion was found in the foreign *atp1 *genes from *P. macrorhiza *lines A and B. In these two lines, a 15 nucleotide (nt) region (positions 298-312) is identical to the native *atp1 *copy from both *P. macrorhiza *and *P. coronopus *but differs from *Cuscuta atp1 *at five sites and from the other foreign *Plantago atp1 *genes at six sites (Figure 3a). The third conversion also involves the foreign *atp1 *gene from *P. macrorhiza *line B and is found only in this sequence. However, unlike the previous example, the converting sequence does not appear to be the native *P. macrorhiza atp1 *gene. Instead, the 157-nt, putative conversion region (positions 582-738) is most similar to *P. sericea *(Figure 3a), a distantly related species of *Plantago *in a different subgenus. Phylogenetic analysis places this converted region strongly with native *atp1 *genes from *P. lanceolata *and *P. sericea *(89% bootstrap support), specifically as sister to *P. sericea*, whereas the unconverted sections of this sequence group in the originally identified position with the foreign *atp1 *copy of *P. macrorhiza *line A (Figure 3b).

Although gene conversion was detected for *atp1 *but not *atp6 *or *matR*, conversion is not the cause of the different phylogenetic relationships observed among the foreign copies of *atp1 *compared to the other two genes (Figure 1). When we removed all converted regions from the *atp1 *data, the resulting phylogenetic tree (Additional File 4c) had the same topology as seen in the initial analysis (Figure 1).

### Mitochondrial location of horizontally transferred genes

In previous studies [23,25,30,31], a mitochondrial location for horizontally acquired genes was inferred by finding sites of RNA editing in their transcripts. However, expression was not detected for any of the foreign copies identified here (Figure 2), so we took advantage of the fact that the mitochondrial, plastid and nuclear genomes differ widely in copy number in plant cells to determine the compartmental location of these unexpressed pseudogenes. The plastid genome is generally present in hundreds to thousands of copies per leaf cell, the mitochondrial genome in tens to hundreds of copies per cell and the nuclear genome usually only in two copies per cell [65,66]. As a precedent, we point out that the differential copy number between nuclear and mitochondrial genomes has enabled an accurate estimation of genome location by Southern blot hybridization (for example, [54,55]). Here we use quantitative (q) PCR instead of blots for two reasons: the intrinsically quantitative nature of qPCR and the opportunity to factor out sequence divergence (by using primers that perfectly match all sequences; Southern blot studies can be confounded by differential substitution rates, both between mitochondrion and nucleus, and among plant lineages).

**Figure 4:**
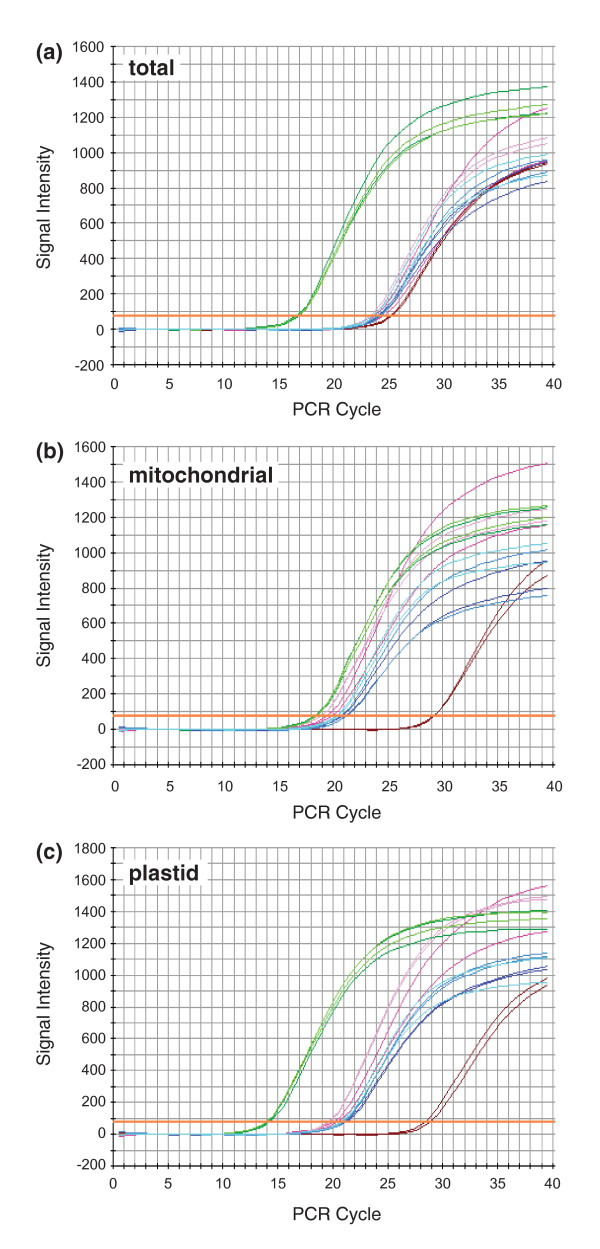
**Genomic location of foreign genes**. Quantitative polymerase chain reaction was used to amplify five loci of known genomic origin [plastid *rbcL *(light green) and *trnL*/*trnF *(dark green), mitochondrial *atp1 *(light pink) and *matR *(dark pink) and nuclear *phyA *(maroon)] and three foreign genes of uncertain provenance [*atp1 *(light blue), *atp6 *(medium blue) and *matR *(dark blue)]. All eight reactions were run in duplicate using as templates, either total genomic DNA (A), mitochondrial-enriched DNA (B) or plastid-enriched DNA (C) isolated from *Plantago coronopus *line C.

QPCR was performed with primers designed to known loci in the nuclear (*phyA*), mitochondrial (native *atp1 *and *cox1*) and plastid (*rbcL *and intergenic spacer *trnL*/*trnF*) genomes, as well as to the horizontally acquired pseudogenes (*atp1, atp6 *and *matR*). All eight of these regions were amplified from *P. coronopus *line C using total genomic DNA, mitochondrial-enriched DNA and plastid-enriched DNA and the relative intensities were compared (Figure 4). The known mitochondrial, plastid and nuclear genes display patterns generally consistent with expectation, although mitochondrial DNA copy number in *P. coronopus *may be relatively low compared to other plants. In the total DNA preparation (Figure 4a), the plastid genes are in the highest copy (lowest cycle number), the nuclear gene is in the lowest copy and the mitochondrial genes are at an intermediate level. In the mitochondrial-enriched preparation (Figure 4b), the plastid DNA is still in the highest copy and the nuclear DNA remains the lowest but the mitochondrial DNA is shifted substantially to the left relative to the other two genomes, indicating that considerable enrichment for mitochondrial DNA was indeed achieved. In the plastid-enriched preparation (Figure 4c), all three genomes are well-separated and, importantly, this preparation appears to be substantially enriched in both plastid *and *mitochondrial sequences relative to nuclear ones.

For all three preparations of plant DNA, the three foreign genes amplify at rates comparable to the two native mitochondrial genes examined. This is most evident in the plastid-enriched DNA which provides the greatest separation among mitochondrial, plastid and nuclear genes, but it is also consistent in the other two DNA preparations. The clear and consistent association of the three foreign *atp1 *genes with mtDNA in terms of copy number indicates that, in *P. coronopus *line C at least, they are located in the mitochondrion, most likely integrated into the mitochondrial genome.

### Gene transfer was DNA mediated

There are a number of possible mechanisms of horizontal transfer, some of which include a stage involving RNA. In order to test whether transfer occurred through an RNA intermediate, we looked for evidence of C-to-U RNA editing that occurs at multiple positions in nearly all mitochondrial protein-gene transcripts from flowering plants [47-53]. If an RNA intermediate is involved, there must be a point at which the transcript is reverse transcribed back into cDNA. Assuming that reverse transcription occurs after RNA editing of the transcript, the edited positions containing U in the RNA will be reverse transcribed to T residues in the cDNA. Thus, an RNA-mediated gene transfer event should contain T residues at most, or all, sites of RNA editing in the original donor gene [34,35], whereas a transfer of genomic DNA should retain C residues at the edit sites.

**Figure 5:**
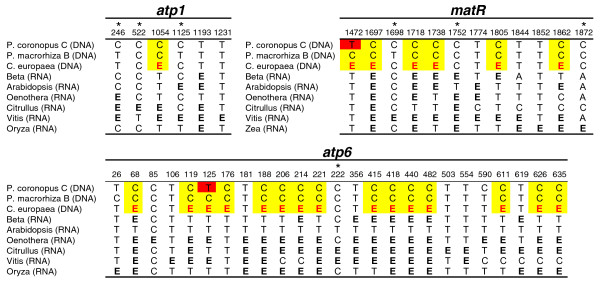
**Status of RNA edit sites in the foreign genes**. Shown are those nucleotides in foreign *Plantago *genes that are known or predicted to be edited in at least one of the other species listed. Edited cytidines are marked with the bold letter E and are shown in black if taken from previous experimental analyses and in red if predicted. Cytidines in *Plantago coronopus *line C and *P. macrorhiza *line B that are homologous to the predicted edit sites in *C. europaea *are highlighted in yellow, whereas thymidines are highlighted in red. Edit sites at third positions are marked with an asterisk.

A comparison of editing positions in *Cuscuta *to homologous positions in the *Plantago *HGT copies shows that there is no evidence for a massive C to T conversion in *Plantago *that would be expected if the transfer(s) was RNA mediated (Figure 5). Known edit sites in six species were used to predict edit sites in *C. europaea *using the RNA-editing prediction tool PREP-Aln [67]. In total, 26 edit sites are predicted for *C. europaea*: 1 in *atp1*, 15 in *atp6 *and 10 in *matR*. Of these, 22 are in regions also sequenced for the HGT copies from *P. coronopus *and *P. macrorhiza*. *P. macrorhiza *has a cytidine at all 22 positions and *P. coronopus *has 20 cytidines and only two thymidines. Thus, there is essentially no evidence that the transfer event proceeded through an RNA step. Although this analysis cannot completely rule out the possibility that reverse transcription occurred from unedited transcripts, this seems unlikely to have independently occurred for all three genes because the majority of edited sites are found fully edited in surveys of the mitochondrial transcript pool [47,50,51,53].

## Discussion

### The process of horizontal gene transfer between plants

The finding, for three different genes, of a monophyletic *Plantago *pseudogene group within the parasitic genus *Cuscuta *indicates that a species of *Cuscuta *transferred genetic material to the common ancestor of *P. coronopus, P. macrorhiza *and *P. subspathulata *(Figure 1). Of the roughly 220 species in genus *Plantago*, these three species (and the unsampled *P. crypsoides*) are more closely related to one another than to all other members [59,60]. As *Cuscuta*-like copies of *atp1, atp6 *and *matR *were not isolated from any species from the remaining major clades of subgenus *Coronopus *or from any member of the other subgenera, the timing of transfer can be assigned to the period prior to the radiation of *P. coronopus*, *P. subspathulata *and *P. macrorhiza *and subsequent to the split between their common ancestor and the rest of subgenus *Coronopus*. Thus, this transfer event is very recent, certainly within the last few million years, given that the entire genus has been estimated to be only 5-17 million years old [56,60]. As suggested previously [27], HGT was probably enabled by direct, physical contact between *Cuscuta *and *Plantago *during a parasitic interaction. Indeed, *Plantago *is known to be at least occasionally parasitized by *Cuscuta *[68,69]. Furthermore, many species of *Cuscuta *are native to the same regions in the Mediterranean as *P. coronopus, P. subspathulata *and *P. macrorhiza *[59,70], suggesting that the HGT event occurred in this area.

The retention of cytidines in *P. coronopus *and *P. macrorhiza *at edit sites in *Cuscuta *and related species (Figure 5) strongly points to DNA as the transferred genetic material rather than RNA or cDNA. This finding, plus the phylogenetic distribution mentioned above, strongly suggests that all three genes were acquired together in a single transfer event. PCR was not performed to investigate whether these genes are adjacent in any *Cuscuta *or *Plantago *genome. However, a survey of the complete mitochondrial genomes from 17 seed plant species in GenBank revealed that *atp1, atp6 *and *matR *are usually separated by tens to hundreds of kb and are never clustered together to the exclusion of any other genes. The smallest segment in which these three genes cluster in any of the sequenced seed plants is 39 kb in *Triticum*. Six other genes also cluster in this *Triticum *genomic segment. Thus, although it is possible that these three genes were adjacent in the donor *Cuscuta *mitochondrial genome, it is more likely that the transfer involved a large DNA fragment containing additional genes and non-genic regions of the mitochondrial genome which, typically, are very large in plants with 80%-90% of the genome consisting of intergenic spacer DNA. It is even possible that an entire *Cuscuta *mitochondrial genome was transferred. Although we did not detect any additional HGT genes in the PCR-based survey, it should be pointed out that we recovered data for only 10 *Plantago *genes out of the 30-40 typically present in plant mitochondrial genomes and that the survey did not examine intergenic regions. The possibility remains that additional transferred sequences persist to this day in one or more of these *Plantago *species or they may have been historically present but have been lost at some point after transfer.

Was the DNA transferred in a naked form or packaged inside a mitochondrion, virus, phytoplasma or some other agent? It seems unlikely that a large, unassisted and unprotected fragment of DNA could successfully traverse the gauntlet of obstacles in the way of successful transfer and, therefore, a packaged transfer seems more likely. Whether the vehicle was a bacterium, virus, mitochondrion or something else remains uncertain. Complete mitochondrial sequencing is underway and should provide a significant insight into the mechanisms of transfer between *Plantago *and *Cuscuta*, including: whether additional mitochondrial genes from *Cuscuta *were transferred; whether the transferred genes cluster in the genome; and whether bacterial or viral sequences are in the vicinity of the transfer.

### The consequences of horizontal gene transfer between plants

In order for a horizontal transfer event to be successful, DNA must not only be transmitted from donor to recipient but also integrated into the recipient's genome (in meristematic tissue in the case of plants) and subsequently fixed throughout the population. The qPCR results clearly indicate a mitochondrial location for those foreign genes examined (Figure 4) but the phylogenetic incongruence between *atp1 *and the other two genes (Figure 1) and the large difference in synonymous substitution rate in the horizontal copies relative to functional mitochondrial genes (Table 1) defy a simple scenario of mitochondrial integration followed by strictly vertical inheritance (see the next section).

Regardless of the point of integration, the three *Cuscuta*-derived genes in *Plantago *have clearly degraded into pseudogenes soon after acquisition by HGT. The optimal value for ω was < 1 in all pairwise comparisons (Table 1) suggesting that the transferred genes may have been under functional constraint for some period of time before they lost function. Alternatively, ω < 1 may simply be a consequence of gene conversion which would be expected to reduce ω at positions converted by functional genes. The three conversion events detected in the *atp1 *pseudogenes were excluded from the ω calculations performed here but it is certainly possible that additional small-scale events remain undetected in the pseudogene sequences. Despite these uncertainties, it is clear that these transferred genes did eventually degrade into pseudogenes and probably did not provide any selective advantage to the *Plantago *recipients. Thus, fixation of the transfer presumably occurred by genetic drift.

From a broader perspective, the detection of gene conversion in this study suggests that HGT among plant mitochondrial genes may have greater evolutionary significance than currently realized. Two of three cases of putative gene conversion involving foreign pseudogenes provide straightforward evidence of conversion between native and foreign genes that were probably located in the same organism and genome. Although in both cases it seems likely that the foreign pseudogenes were converted by native functional homologs, the alternative scenario of native functional genes being converted by foreign genes is now certainly plausible (and indeed has just been reported [31]) and could lead to increased genetic diversity of the recipient mitochondrial genome and, possibly, even adaptive benefits. The third example of apparent gene conversion is much less straightforward in interpretation and probably more complicated in derivation, involving the conversion of the foreign *atp1 *gene of one of two lines of *P. macrorhiza *examined by a *P. sericea*-like gene. Taking the alignment (Figure 3a) and phylogenetic analysis (Figure 3b) of different parts of this gene at face value, it would appear that *P. macrorhiza *line B acquired *atp1 *by HGT twice, with an *atp1 *gene acquired from the *P. sericea *lineage converting a short central segment of the *atp1 *pseudogene acquired from *Cuscuta*. Alternatively, this segment in the foreign copy of *atp1 *may have been converted by a native copy of *atp1 *that is present in the nucleus of *P. macrorhiza*, with the mutation rate heterogeneity responsible for the apparent phylogenetic conflict. That is, the synonymous substitution rate in the *P. macrorhiza *mitochondrial lineage clearly has been much higher than in *P. sericea *and may also be high compared to the nuclear rate in *P. macrorhiza *(Figure 1[56]). Therefore, the stronger similarity of this converted region to *P. sericea *may simply reflect a greater retention of shared ancestral characters in the *P. sericea *mitochondrial copy and the putative, native *P. macrorhiza *nuclear copy relative to the native *P. macrorhiza *mitochondrial copy.

The detection of three separate conversion events in this study illustrates the under-appreciated proclivity of non-identical segments of DNA to intermix in plant mitochondrial genomes. The often surreptitious nature of these events is underscored by the fact that two of these converted regions, which are present in sequences generated for the initial HGT report between *Cuscuta *and *Plantago *[27], went undetected for several years. Reports documenting the generation of chimeric mitochondrial genes through recombination with horizontally acquired genes are becoming increasingly common [23,30,31]. Furthermore, the mitochondrial *atp1 *gene was recently shown to have been converted by homologs of plastid origin on a number of occasions during angiosperm evolution [71,72]. Gene conversion between nuclear and mitochondrial sequences has yet to be reported, but the converted region in *P. macrorhiza *B may be the result of just such an interaction.

### Causes of rate heterogeneity and phylogenetic incongruence within clades of foreign *Plantago *genes

Numerous factors are known to cause substitution rate variation or phylogenetic incongruence. The combination of both phenomena in all three horizontally acquired genes suggests that they may be causally linked in *Plantago*. One explanation is that we are comparing paralogous or xenologous pseudogenes rather than orthologs, resulting in phylogenetic incongruence, potentially accompanied by longer branch lengths than might otherwise be expected. These copies could result from duplication of the foreign genes after an initial horizontal transfer event or from multiple independent transfer events from the same or different *Cuscuta *donors. Differential gene loss or differential recovery by PCR could then explain the phylogenetic incongruence in different lines. As only one pseudogene copy was obtained from each examined line, differential loss would have to remove all but one pseudo-copy in each lineage, which is perhaps unlikely. Differential PCR recovery could occur because a given gene copy sustained mutations that make it a poor substrate for amplification relative to the copy recovered and/or is present in much lower copy number. This would be the case if the recovered copy were on the major mitochondrial genome form and the other copy were present as either a highly sub-stoichiometric form (such 'sublimons' are well known to occur in plant mitochondria [73]) or, via intracellular gene transfer, in the nuclear genome [65,66].

On top of these potentially confounding processes is the possibility that substitution rates vary spatially and/or temporally among the different genes and species lines. This is suggested in comparisons of branch lengths between the *atp6 *and *matR *foreign sequences that otherwise share the same topology (Figure 1) and in the overall higher level of divergence in foreign genes relative to native (Figure 1, Table 1). Regional variation in mitochondrial substitution rate, although not widely observed in plants, has been reported in *Silene *[74,75]. At present, there is insufficient data to know whether mutation rates might vary sharply within *Plantago *mitochondrial genomes. Trans-compartmental rate variation is widely found in plants, with nuclear genomes generally evolving significantly faster than mitochondrial genomes [76]. The situation is much more complicated in *Plantago*, however, where highly elevated mitochondrial rates have been documented in large portions of its phylogeny, but with subsequent marked decreases in rates in most recently derived lineages, including, probably, the *P. coronopus *clade ([56]; JPM and JDP, unpublished data). Furthermore, almost nothing is known about nuclear mutation rates in *Plantago*, especially in the *P. coronopus *clade [the only nuclear sequence available for this group is ITS (Figure 1) which is problematic because it is non-coding and subject to concerted evolution]. These many potential sources of rate variation could greatly exacerbate the phylogenetic uncertainties caused by extra pseudo-copies.

## Conclusions

We provide here the strongest evidence yet that multiple mitochondrial genes can be transferred via a single HGT event and we demonstrate that transfer occurred via a DNA intermediate. Given that this HGT probably involved a large segment of mitochondrial DNA, transfer via naked DNA or a viral vector is deemed unlikely. Rather, transfer probably involved an organismal intermediate, either a relatively large vectoring agent (for example, a bacterium, fungus or insect) or the donor plant itself through a direct fusion of native and foreign mitochondria (with foreign mitochondria spread perhaps via illegitimate pollination, grafting or wounding). Also, our results provide clear evidence for mitochondrial integration of transferred genes. The detection of three separate gene conversions between co-resident foreign and native mitochondrial homologs suggests that transferred genes may be evolutionarily important in generating mitochondrial genetic diversity. The complex relationships within each lineage of transferred genes imply a surprisingly complicated history of these genes in *Plantago *subsequent to their acquisition via HGT, with this history likely involving some combination of additional transfers (including intracellular transfer), gene duplication and differential loss and mutation-rate variation. Resolving the relative contributions of gene duplication, horizontal and/or intracellular transfer and substitution rate variation to the origin and diversification of these foreign genes will probably require complete mitochondrial and nuclear genome sequencing from multiple individuals of the *P. coronopus *clade and related taxa.

## Methods

### Sources of materials

Voucher information and source of seeds and DNAs for all *Plantago *species used in this study are provided in Additional File 3. Seeds were germinated and grown to maturity in the Beadle Center Greenhouse (University of Nebraska-Lincoln). Additional DNA samples used in the study to generate sequence data were available from previous studies [77-79].

### Nucleic acid isolation

Total genomic DNA and RNA were isolated from fresh leaves of greenhouse-grown plants using DNeasy and RNeasy Plant Mini Kits (QIAGEN, CA, USA). At least two DNA extractions were performed on separate occasions for each plant grown at the University of Nebraska-Lincoln (Additional File 3).

Plastid- and mitochondrial-enriched DNA was isolated from *P. coronopus *line C using the differential-centrifugation portion of the protocol described in detail by Palmer [80] and summarized here. Fifty grams of fresh, young leaf tissue were homogenized in a Waring blender and then filtered through four layers of cheesecloth followed by one layer of Miracloth. The filtrate was centrifuged at 500 × g for 10 min to pellet nuclei and cellular debris. The suspension was transferred to a new bottle and centrifuged at 2500 × g for 15 min. The pellet from this spin was retained as the chloroplast-enriched fraction and the supernatant was centrifuged at 12000 × g for 20 min. The pellet from this last spin was retained as the mitochondrial-enriched fraction. DNA was isolated from the plastid- and mitochondrial-enriched pellets using the DNeasy Plant Mini Kit (QIAGEN).

### Polymerase chain reaction

Conventional PCR was performed on a PTC-100 (MJ Research, Waltham, CT, USA) or PTC-0220G (Bio-Rad, CA, USA) thermocycler using standard reaction conditions (initial step of 3 min at 94°C; 35 cycles of 30 s at 94°C, 45 s at 48°C, 90-120 s at 72°C; final step of 10 min at 72°C). For the HGT survey, a previously developed set of 96 primer pairs covering most known mitochondrial protein and rRNA genes from plants [25] was used on total DNA from *P. coronopus *line B and *C. gronovii*. Preliminary phylogenetic analyses were performed to screen for potential cases of HGT. For all potentially foreign genes, homologs were PCR-amplified from additional species to increase taxon sampling. In addition, putatively native copies were amplified from total DNA using copy-specific primers designed to avoid amplification of the foreign homologs. All PCR products were purified and sequenced on both strands at the Indiana Molecular Biology Institute (Indiana University, Bloomington, USA) or the High-Throughput Genomics Unit (University of Washington, Seattle, USA). All *Plantago *sequences reported in this study were amplified and sequenced at least twice from each DNA extraction. All newly generated sequences were deposited in GenBank (accessions HQ593736-HQ593805 and HQ593815-HQ593837). Additional DNA sequences used in this study were acquired from GenBank. Full lists of sequences used are provided in Additional File 2.

QPCR assays were performed on a iCycler iQ thermocycler (Bio-Rad) using iQ SYBR Green Supermix (Bio-Rad) according to manufacturer instructions. QPCR primers were designed using the online RealTimePCR Tool with default parameters (Integrated DNA Technologies, IA, USA). Primers were designed to amplify eight loci from *P. coronopus *line C: five putatively native loci [mitochondrial *atp1 *(124 bp product) and *cox1 *(140 bp), plastid *rbcL *(82 bp) and *trnL/trnF *(135 bp) and nuclear *phyA *(137 bp)] and the three putatively foreign genes [*atp1 *(73 bp), *atp6 *(77 bp) and *matR *(135 bp)]. Designed primers averaged 19 bp in length (range 18-22 bp), 49% GC content (range 43%-53%) and 60°C melting temperature (range 59°-61°C). For each 20 μL reaction, 10 ng of DNA (plastid-enriched, mitochondrial-enriched, or total genomic) from *P. coronopus *line C was added. Each reaction was run in duplicate. To ensure that all primer combinations amplified efficiently, a four-step series of five-fold dilutions was performed starting from 25 ng total genomic DNA and amplification efficiency was calculated using iCycler software. All products demonstrated an efficiency >90%.

RT-PCR was performed as described previously [79] and summarized here. Purified RNA from *P. coronopus *line C and *P. macrorhiza *line B was treated with RNase-free DNase I (Fermentas) and then converted to cDNA using M-MuLV reverse transcriptase (Fermentas) and random hexamers (Fermentas) according to manufacturer instructions. In order to test for the presence of genomic DNA contamination, a negative control reaction excluding reverse transcriptase was run in parallel with the reaction that included reverse transcriptase. Primers and PCR conditions used for RT-PCR were identical to those used for conventional PCR.

### Molecular evolutionary analyses

Sequence alignments were constructed with MUSCLE version 3.7 [81] and manually adjusted when necessary using BioEdit version 7.0.9 [82]. For pseudogenes with frameshifting indels, the sequences were restored to their ancestral reading frames by comparison with functional gene copies from closely-related sequences. This was necessary for calculations of synonymous and non-synonymous sequence divergence. Poor-quality regions of the alignments were excluded using Gblocks version 0.91b [83] with relaxed parameters including the minimum number of sequences for a flank position (b2) set to 50%, minimum block length (b4) set to 5, and maximum number of species with gaps (b5) set to 50%.

For some analyses as indicated in the text, predicted sites of C-to-U RNA editing were eliminated by converting them to T in the data sets. In order to predict edit sites, data set sequences were first aligned to published cDNA sequences from *Arabidopsis thaliana, Beta vulgaris, Citrullus lanatus, Vitis vinifera, Oenothera berteriana *(for *atp6 *and *matR*) or *O. biennis *(for *atp1*) and *Oryza sativa *(for *atp1 *and *atp6*) or *Zea mays *(for *matR*). Edit sites were predicted in the data set sequences by comparison to the cDNA sequences using PREP-Aln [67] with a cutoff score of 0.2, and all predicted sites were converted to T.

Phylogenetic analyses were performed using the maximum likelihood (ML) approach as implemented in PhyML version 3.0 [84]. For each analysis, the general time reversible (GTR) substitution model and subtree pruning and regrafting (SPR) branch-swapping was used. A gamma distribution with four rate categories and the proportion of invariable sites were estimated during the analysis. Each analysis was run five times starting from different randomized tress. Support for the ML topology was evaluated by bootstrapping with 100 ML replicates.

Pairwise levels of non-synonymous (dN) and synonymous (dS) divergence were calculated with MEGA version 4.0.2 [85]. The Nei-Gojobori method was used with a Jukes-Cantor correction for multiple hits and pairwise deletion of gaps. Standard errors for the pairwise estimates were calculated using the bootstrap method with 500 replicates. Edit site effects were eliminated from the analyses by coding all predicted sites as a T in the data sets. Effects of *atp1 *gene conversion were eliminated from the analysis by removing the affected codons from the data set.

Recombination was detected by OnePop in the OrgConv package [64]. When the length of the detected recombinant segment was longer than 100 nucleotides, phylogenetic trees were reconstructed for both the recombinant region and the remaining sequence using PhyML as described above, and incongruence between the regions was examined using the approximately unbiased (AU) test [86]. Detected recombinant segments were required to have a *P*-value < 0.001 to be considered significant, and when longer than 100 nucleotides, the segment was required to have a *P*-value < 0.05 using the AU test.

## Abbreviations

cDNA: complementary DNA; C-to-U: cytidine to uridine; HGT: horizontal gene transfer; ITS: internal transcribed spacer; ML: maximum likelihood; nt: nucleotide; numt: nuclear-encoded DNA of mitochondrial origin; PCR: polymerase chain reaction; qPCR: quantitative PCR; rRNA: ribosomal RNA; RT-PCR: reverse transcription PCR.

## Authors' contributions

JPM, SS, WH, JSG, KJ and DA performed the research. JPM, SS, WH and JDP designed the study and analysed the results. JPM, WH and KJ prepared the figures and tables. JPM and JDP drafted the manuscript and oversaw the study. All authors read and approved the final manuscript.

## Supplementary Material

Additional file 1 **Alignments**. Nucleotide sequence alignments for *atp1*, *atp6 *and *matR *for selected taxa. Click here for file

Additional file 2 **GenBank accession numbers**. Accessions are provided for all newly generated as well as previously available sequences used in this study. Click here for file

Additional file 3 **Sources of material**. Sources, type of material, and voucher information for *Plantago *species used in this study. Click here for file

Additional file 4 **Additional phylogenetic analyses**. The *atp1*, *atp6 *and *matR *data sets were re-evaluated in several alternative ways. Click here for file

Additional file 5 **Tests of alternative tree topologies**. Results of Shimodaira-Hasegawa Tests for comparisons of alternative phylogenetic hypotheses. Click here for file
