# Supplementary material for: Horizontal acquisition of multiple mitochondrial genes from a parasitic plant followed by gene conversion with host mitochondrial genes
Source: BMC Biol. 2010 Dec 22;8:150. doi: 10.1186/1741-7007-8-150 (PMC3022774; doi:10.1186/1741-7007-8-150)
Supplement: Additional file 1 — Alignments. Nucleotide sequence alignments for atp1, atp6 and matR for selected taxa. [file 1741-7007-8-150-S1.PDF]

Additional File 1 – **Nucleotide sequence alignments.**

Alignments for (A) *atp1*, (B) *atp6*, and (C) *matR* for selected taxa are shown. Nucleotides are denoted as dots when identical to the reference sequence from *Nicotiana*. The sequences resulting from gene conversion are highlighted as in figure 4, except that the conversion of the mitochondrial *atp1* gene by the plastid *atpA* gene [71] is also shown and is highlighted in yellow. Positions that contain an 'x' indicate insertions into the pseudogene sequences to correct reading frames after a frameshifting indel.

Additional File 1A - *atp1* alignment

```
                                 10         20         30         40         50         60         70         80         90        100
                         ....|....| ....|....| ....|....| ....|....| ....|....| ....|....| ....|....| ....|....| ....|....| ....|....|
Nicotiana                ATCGGTCGAG TGGTCTCAGT TGGAGATGGG ATTGCACGTG TTTATGGATT GAACGAGATT CAAGCTGGGG AAATGGTTGA ATTTGCCAGC GGTGTGAAAG
Convolvulus              .......... .......... .......... .......... .......... .......... .......... .......G.. .......... ..........
Dinetus                  .......... .......... .......... .......... .......... .......... .......... .......G.. .......... ..........
C. europaea              .......... .......... .......... .......... .......... .......... .......... .......G.. .......... ..........
P. coronopus A pseudo                                  ... ....T..... .......... ....A..... ...------- -......A.. ....A..... .A........
P. coronopus B pseudo     ......... ....T...A. .A........ ....T..... .......... ....A..... ...------- -......A.. ....A..... ..........
P. coronopus C pseudo                    ...-. .A......A. ....T..... .......... ....A..... ...------- -......A.. ....A..... ..........
P. coronopus D pseudo                                   ... ......---- -----..... .......... .G........ .......A.. .......... ..........
P. macrorhiza A pseudo    ......... ........T. .......... .......... ...G...... .......... .......... .......A.. .......... ..........
P. macrorhiza B pseudo                       . .......... .......... ...G...... .......... .......... .......A.. .......... ..........
P. subspathulata pseudo   ......... ....T...A. .A........ ....T..... .......... ....A..... T..------- -......A.. ....A..... ..........
Jovellana                 .......C. .......... .........T .......... .......... .......... .......... .......... .......... ..........
Streptocarpus             ......... .......... .........T .......... .......... .......... .......... .......A.. .......... ..........
Mimulus                  .......... .......... .........T .......... .......... .......... .......... .......... ....T..... ..........
Aragoa                    .T....... .......... .........  .......... .......... A..T...... ........A. .......... ....T..... ..........
Digitalis                .......... .......... .........T .......... .......... .......... .......... .......... ....T..... ..........
Globularia                             ....T.. .........T .......... .......... .......... .......... .......... ....T..... ..........
P. lanceolata            .......... .......T.. C........T ........G. .......... A..G...... ........C. .......C.. .......... ..G..C....
P. sericea                ......... .A.....C.. C........T ........G. .......... A..G...... ........A. .......C.. .........T ..G.......
P. crassifolia            ......... .C.....T.. .........T ..C...A.G. .......... A..G...... .......... .......G.. ........C. ..C..C....
P. coronopus C funct                                    T ..C...A.G. .......... A..G...... .....G..A. .......G.. ........C. ..C..C....
P. macrorhiza B funct                        . ...G.....T ..C...A.G. .......... A..G...... .....G..A. .......G.. ........C. ..C..C....

                                110        120        130        140        150        160        170        180        190        200
                         ....|....| ....|....| ....|....| ....|....| ....|....| ....|....| ....|....| ....|....| ....|....| ....|....|
Nicotiana                GAATAGCCTT GAATCTTGAG AATGAGAATG TAGGGATTGT TGTCTTTGGT AGTGATACTG CTATTAAAGA AGGAGATCTT GTCAAGCGCA CTGGATCTAT
Convolvulus              .......... .......... .......... .......... .......... .......... .......G.. .......... .......... ...N......
Dinetus                  .......... .......... .......... .......... .......... .......... .......G.. .......... .......... ..........
C. europaea              .......... .......... .......... .......... .......... .......... .......G.. .......... ..A....... ..........
P. coronopus A pseudo    .......T.. ....T..... .......... .......... .........A ........C. .....C.... .......... ........G. .C........
P. coronopus B pseudo    ....C..T.. ....T..... .......... .......... .........A ........C. .....C.... .......... ......T.G. ..........
P. coronopus C pseudo    ....C..T.. ....T..... .......... .......... .........A ........C. .....C.... .......... ......T.G. ..........
P. coronopus D pseudo    .........C ....T..... .......... .......... .......... .......... .......G.. .......... ..A..A.... ..........
P. macrorhiza A pseudo   .........C ....T..... .......... .......... .......... G......... .......G.. .......... ..A..A.... ..........
P. macrorhiza B pseudo   .........C ....T..... .......... .......... .......... G......... .......G.. .......... ..A..A.... ..........
P. subspathulata pseudo  .......T.. ....T..... .......... .......... .........A ........C. .....C.... .......... ........G. ..........
Jovellana                .......... C......... .......... .......... .......... .......... .......... .......... .......... ..........
Streptocarpus            .......... A......... .......... .......... .......... .......... .......... .......... .......... ..........
Mimulus                  .......... A......... .......... .......... .......... .......... .......... .......... .......... ..........
Aragoa                   ....C..... A......... .......... .......... .......... .......... .......... .......... .......... ..........
Digitalis                .......... A......... .......... .......... .......... .......... .......... .......... .......... ..........
Globularia               .......... A......... .......... .......... .......... .......... .......... .......... .......... ..........
P. lanceolata            ....G..... A.....A... .......... .T..C..... C........G ........G. .....C.... .......... ..G....... ..........
P. sericea               ....C..... A.....G... .....A.... ....T..... C......... ........G. .......... .......... ..G....... .A........
P. crassifolia           ....C..... A.....C..A .......... ....C..... G........A ........C. .....C.... .......... ..A.....T. .C...G.C..
P. coronopus C funct     ....C..T.. A.....A..A .......... .......... .........A ........C. .....C.... .......... ........G. .C.....C..
P. macrorhiza B funct    ....C..T.. A.....A..A .......... .......... .........A ........C. .....C.... .......... ........G. .C.....C..

                                210        220        230        240        250        260        270        280        290        300
                         ....|....| ....|....| ....|....| ....|....| ....|....| ....|....| ....|....| ....|....| ....|....| ....|....|
Nicotiana                TGTGGATGTT CCTGCGGGAA AGGCTATGCT AGGGCGTGTG GTCGATGGCT TGGGAGTACC TATTGATGGA AGGGGGGCTC TAAGCGATCA CGAGCGAAGA
Convolvulus              .......... .......... .......... .N........ .....C.C.. .......... .......... ..A....... .......... ..........
Dinetus                  .......... .......... .......... .......... .....C.C.. .......... .......... ..A....... .......... ..........
C. europaea              .......... .......... .......... ...A...... .....C.CG. .......... .......... ..A....... .......... T.........
P. coronopus A pseudo    ...C..A... .......... ..A..G.... ...A...... .....C.CG. ..A....... .......... ..A....... .C.A...G.. ..........
P. coronopus B pseudo    ...C..A... .......... ..A..G.... ...A...... .....C.CG. C.A....... ...--..... ..AA...... .C.A...G.. T.C.......
P. coronopus C pseudo    ...C..A... .......... ..A..G.... ...A...... .....C.CG. C.A....... ...--..... ..AA...... .C.A...G.. T.C.......
P. coronopus D pseudo    ...C...... ..C....... G......... ...A...... .....C.CA. ..A....... C...A..T.. ..A....... .......G.. .....A....
P. macrorhiza A pseudo   .......A.. .......... .......... ...A...... .....C.CA. ..A....... ....A..T.. ..A....... .......G.. .......C..
P. macrorhiza B pseudo   .......A.. .......... .......... ...A...... .....C.CA. ..A....... ....A..T.. ..A....... .......G.. .......C..
P. subspathulata pseudo  ...C..A... .......... ..A..G.... ...A...... .....C.CG. ..A.....T. .......... ..A....... .C.A...G.. ..........
Jovellana                .......... .......... .......... .......... .....C.C.. .......... .......... ..A....... ......C... ..........
Streptocarpus            .......... .......... .......... .......... .....C.C.. .......... .......... ..A....... .......... ..........
Mimulus                  .......... .......... .......... .......... .....C.C.. .......... .......... ..A....... ......C... ..........
Aragoa                   .......... .......... .......... .......... .....C.C.. .......... .......... ..A....... ......C... ..........
Digitalis                .......... .......... .......... .......... .....C.C.. .......... .......... ..A....... ......C... ..........
Globularia               .......... .......... .......... .......... .....C.C.. .......... .......... ..A....... ......C... ..........
P. lanceolata            ...C...... .......... ..A....... ...A.....A .....C.... .A..CC.T.. .......... .AA....... ..G.A.C... ......C...
P. sericea               ...C...... .....A.... .......... ...C..G..A .....C..A. .....C.T.. .......... .AA....... ..G.A.C... ......C...
P. crassifolia           ...C...... .....C.... .......... ...AA.G..C .....C..A. ....CC.G.. .......... .AA...C.C. .GG.A.C... ......CC..
P. coronopus C funct     ...C...... ..C....... G......... ...AA.G..C .....C..A. .....C.C.. .......... .AA...C.C. .CG.A.C... .......C..
P. macrorhiza B funct    ...C...... ..C....... G......... ...AA.G..C .....C..A. .....C.C.. .......... .AA...C.C. .CG.A.C... .......C..

                                310        320        330        340        350        360        370        380        390        400
                         ....|....| ....|....| ....|....| ....|....| ....|....| ....|....| ....|....| ....|....| ....|....| ....|....|
Nicotiana                CGTGTCGAAG TGAAAGCC-- ----CCTGGT ATTATTGAAC GTAAATCT-- ----GTGCAC GAGCCTATGC AAACAGGGTT AAAAGCGGTA GATAGCCTGG
Convolvulus              .......... ........-- ----.....G .......... ........-- ----...... .......... .......... .......... ..........
Dinetus                  .......... ........-- ----.....G .......... ........-- ----...... .......... .......... .......... ..........
C. europaea              .......... ........-- ----.....G .......... ........-- ----...... .......... .......... .......... ..........
P. coronopus A pseudo    ......A... ........-- ----.....G ...T...... ........GT CTxx...... .......... .......... T......... ....A.....
P. coronopus B pseudo    ......A... ........-- ----.....G ...T...... ........GT CTxx...... .......... .......... T......... ....A.....
P. coronopus C pseudo    ......A... ........-- ----.....G ...T...... ........GT CTxx...... .......... .......... T......... ....A.....
P. coronopus D pseudo    ......A... .......T-- ----.....G ...T.....T ........-- ----.AA... .....GT... .......... .....T.... ...G...C..
P. macrorhiza A pseudo   A.G..A.... .C.....TCC TGxx.....G ...T...... ........GT CTxx...... .......... .......... .......... ....A.....
P. macrorhiza B pseudo   A.G..A.... .C.....TCC TGxx.....G ...T...... ........GT CTxx...... .......... .......... .......... ....A.....
P. subspathulata pseudo  ......A... ........-- ----.....G ...T...... ........GT CTxx...... .......... .......... T......... ....A.....
Jovellana                .......... ........-- ----.....A .......... ........-- ----...... .......... .......... .......... ..........
Streptocarpus            .......... ........-- ----.....A .......... ........-- ----...... .......... .......... .......... ..........
Mimulus                  .......... ........-- ----.....A .......... ........-- ----...... .......... .......... .......... ..........
Aragoa                   .......... ........-- ----.....A .......... ........-- ----.....T .......... .......... .......... ..........
Digitalis                .......... ........-- ----.....A .......... ........-- ----.....T .......... .......... .......... ..........
Globularia               .......... ........-- ----.....A .......... ........-- ----.....T .......... .......... .......... ..........
P. lanceolata            .......... .C.....G-- ----..A..C .......... .G.....C-- ----.....T .......... ....G..C.. ......C... ......T...
P. sericea               .......... ........-- ----..A..C .......... .G......-- ----..C..T .......... ....T..T.. ......C... .....TT...
P. crassifolia           .......... .C......-- ----.....A .......... .A.....C-- ----..C..T .......... ....C..A.. ......C... ......T...
P. coronopus C funct     A.G..A.... .C.....A-- ----..C..A .......... .A.....C-- ----..A..T .......... ....C..A.. .......... ......T...
P. macrorhiza B funct    A.G..A.... .C.....A-- ----..C..A .......... .A.....C-- ----..A..T .......... ....C..A.. .......... ......T...
```

Additional File 1A - *atp1* alignment

```
                                   410        420        430        440        450        460        470        480        490        500
                            ....|....| ....|....| ....|....| ....|....| ....|....| ....|....| ....|....| ....|....| ....|....| ....|....|
Nicotiana                   TTCCTATAGG TCGTGGTCAA CGAGAACTTA TAATCGGGGA CCGACAAACT GGAAAAACTG CTATTGCTAT CGATACCATA TTAAACCAAA AGCAACTGAA
Convolvulus                 .......... .......... .......... .......A.. .......... ........A. .......... .......... .......... ..........
Dinetus                     .......... .......... .......... .......A.. .......... ........A. .......... .......... .......... ..........
C. europaea                 .......... .......... .......... .......A.. .......... ........A. .......... .......... .......... ..........
P. coronopus A pseudo       .....C...A .......... ...A...... .......A.. ..A....... ......-.C. TG........ .........T .......... .......A..
P. coronopus B pseudo       .....C...A .......... ...A...... .......A.. ..A....... ......-.C. T......... .........T .......... .......A..
P. coronopus C pseudo       .....C...A .......... ...A...... .......A.. ..A....... ......-.C. T......... .........T .......... .......A..
P. coronopus D pseudo       .......G.. ..A....... .......... ....A..A.. .T........ ........AA ......T... .......... ......---- ------.A..
P. macrorhiza A pseudo      .........A .......... ...A...... .......A.. T.A....... ......-.C. T......... .........T .......... ..T....A..
P. macrorhiza B pseudo      .........A .......... ...A...... .......A.. T.A....... ......-.C. T......... .........T .......... ..T....A..
P. subspathulata pseudo     .....C...A .......... ...A...... .......A.. ..C....... ......-.C. T......... .........T .......... .......A..
Jovellana                   .......... ...G...... .......... .......... .........C ........A. .......... .......... .......... .....T....
Streptocarpus               .......... ...G...... .......... .......... .......... ........A. .......... .......... .......... .....T....
Mimulus                     .......... .......... .......... .......... .......... ........A. .......... .......... .......... .....T....
Aragoa                      .......... .......... .......... .......... .......... ........A. .......... .......... .......... .....T....
Digitalis                   .......... .......... .......... .......... .......... ........A. .......... .......... .......... .....T....
Globularia                  .......... .......... .......... .......... .......... ........A. .......... .......... .......... .....T....
P. lanceolata               ....C..... ...A...... ..C.....G. .C.....A.. .........A ........G. .......... ...C..G... G.C....... .AAC.T....
P. sericea                  ....C..... ...G...... .......... .C..T..A.. .........G .......... .C.....A.. ......A... .......... ..AC.T....
P. crassifolia              .G..G..... G.....C... ........C. .......A.. .........C ........C. .......... .......... .......... .....T....
P. coronopus C funct        .G..C..... AA.G..G... A.......C. .C.....A.. .........C ........C. .......... .........T .......... ..ATTT....
P. macrorhiza B funct       .G..C..... AA.G..G... A.......C. .C.....A.. .........C ........C. .......... .........T .......... ..ATTT....

                                   510        520        530        540        550        560        570        580        590        600
                            ....|....| ....|....| ....|....| ....|....| ....|....| ....|....| ....|....| ....|....| ....|....| ....|....|
Nicotiana                   CTCAAGGGCC ACCTCTGAGA GTGAGACATT GTATTGTGTC ------TATG TAGCGATTGG ACAGAAACGC TCAACTGTGG CACAATTAGT TCAAATTCTT
Convolvulus                 .......... T......... .......... .......... ------.... .......... .......... .......... .......... ..........
Dinetus                     .......... T......... .......... .......... ------.... .......... .......... .......... .......... ..........
C. europaea                 .......... T......... .......... .......... ------.... .......... .......... .......... .......... ..........
P. coronopus A pseudo       .........- ------.... .......... A......... ------.... .......... ........A. ....T..... .......... ..--------
P. coronopus B pseudo       .........- ------.... .......... .......... ------.... .......... ........A. ....T..... .......... ..--------
P. coronopus C pseudo       .........- ------.... .......... .......... ------.... .......... ........A. ....T..... .......... ..--------
P. coronopus D pseudo       ..T...T..T TT..T..... A......... .....T...T TATGTT.... .......... ........A. .......... .......... ..........
P. macrorhiza A pseudo      .......... T......... .......... .......... ------.... .......... ........A. .......... .......... ..........
P. macrorhiza B pseudo      .......... T......... .......... .......... ------.... .......... ........A. .......... .C........ GA........
P. subspathulata pseudo     .......... T......... .......... .......... ------.... .......... ........A. ....T..... .......... ..--------
Jovellana                   .....A.... .......... .......... .......... ------.... .......... .......... .......... .......... ..........
Streptocarpus               .....A...T .......... .......... .......... ------.... .......... .......... ........A. .......... ..........
Mimulus                     .....A.... .......... .......... .......... ------.... .......... .......... .......... .......... ..........
Aragoa                      .....A.... .......... ....T..... .......... ------.... .......... .......... ........A. .......... ..........
Digitalis                   .....A.... .......... .......... .......... ------.... .......... .......... .......... .......... ..........
Globularia                  .....A.... .......... .......... .......... ------.... .......... .......... .......... .......... ..........
P. lanceolata               ...C.....T ..T..C.... ......A... .......... ------.... .......... .......... ..C.....C. .C........ G.........
P. sericea                  ...C...... ..T..A.... A...A.G... .......... ------.... .....G.... .........T ........A. .C........ ..........
P. crassifolia              .GAG...... ..G..C.... AG........ ..T....... ------.... .C..C..... .........A ..C..C..T. .......... A........A
P. coronopus C funct        .GA....... .....C..A. AA.....C.. .........T ------.... .T..C..... .........A ..C..C..T. .......... G........A
P. macrorhiza B funct       .GA....... .....C..A. AA.....C.. .........T ------.... .T..C..... .........T ..C..C..T. .......... G........A

                                   610        620        630        640        650        660        670        680        690        700
                            ....|....| ....|....| ....|....| ....|....| ....|....| ....|....| ....|....| ....|....| ....|....| ....|....|
Nicotiana                   TCAGAAGCGA ATGCTTTGGA ATATTCTATT CTTGTAGCAG CCACCGCTTC GGATCCTGCT CCTCTACAAT TTTTGGCCCC ATATTCTGGG TGTGCCATGG
Convolvulus                 .......... .......... ......C... .......... .......... .......... .....G.... .......... .......... ..........
Dinetus                     .......... .......... ......A... .......... .......... .......... .....G.... .......... .......... ..........
C. europaea                 .......... .......... ......A... .......... .......... .......... .....G.... .......... .......... ..........
P. coronopus A pseudo       -.......A. .......... .......... .......... .T......GT .....T.... .....G.... .......T.. .......... ...T....A.
P. coronopus B pseudo       -......... .......... .......... .......... .T......GT .....T.... .....G.... .......T.. .......... ...T....A.
P. coronopus C pseudo       -......... .......... .......... .......... .T......GT .....T.... .....G.... .......T.. .......... ...T....A.
P. coronopus D pseudo       .T........ ...-----.. ......A... .......... .....A.... ....T..... .......... .......T.. .......... ..........
P. macrorhiza A pseudo      .T........ .......... ......C... .......... .T......G. .....T.... .....G.... .......T.. .......... ...T....A.
P. macrorhiza B pseudo      ..C....... .......T.. .......... ..A....T.. .......... C....T.... .....T.... .......T.. C.....G..C ...A.T....
P. subspathulata pseudo     -......... .......... .......... .......... .T......GT .....T.... .....G.... .......T.. .......... ...T....A.
Jovellana                   .......... .......A.. ......C... .......... .......... .......... .....G.... .......... .......... ..........
Streptocarpus               .....G..C. .......A.. ......C... .......... .......... .......... .....G.... .......... .........A ..........
Mimulus                     .......... .......A.. ......C... .......... .......... A......... .....G.... .......... .......... ..........
Aragoa                      .......... .......A.. ......C... .......... .......... .......... .....G.... .......... .......... .....T....
Digitalis                   .......... .......A.. ......C... .......... .......... .......... .....G.... .......... .......... ..........
Globularia                  .......... .......A.. ......C... .......... .......... .......... .....G.... .......... .......... ..........
P. lanceolata               ..C...AAC. ......C... ......C... .......... .......... ...C.....G .....C.... ..C....... C.....G..C ..........
P. sericea                  ..C....... .......... ......C... .......... .G........ C......... .....C.... ..C.C..... C.....G..C .....T....
P. crassifolia              G.......A. .....A.... ......C... ..A....... .......... C.....G... ..C.....G. .......A.. C..C..C..A ..........
P. coronopus C funct        G......... .....A.... ......C... .....C..C. .T........ C........G ..C.....G. .......A.. C..C..C..C ..........
P. macrorhiza B funct       G......... .....A.... ......C... .....C..C. .T........ C........G ..C.....G. .......A.. C..C..C..C ..........

                                   710        720        730        740        750        760        770        780        790        800
                            ....|....| ....|....| ....|....| ....|....| ....|....| ....|....| ....|....| ....|....| ....|....| ....|....|
Nicotiana                   GGGAATATTT CCGCGATAAT GGAATGCACG CATTAATAAT CTATGATGAT CTTAGTAAAC AGGCGGTAGC ATATCGACAA ATGTCATTAT TGTTACGCCG
Convolvulus                 .......... .......... .......... .......... .......... ..G....... .......... .......... .......... ..........
Dinetus                     .......... .......... .......... .......... .......... .......... .......... .......... .......... ..........
C. europaea                 .......... .......... .......... .......... .......... ........G. .......G.. .......... .......... ..........
P. coronopus A pseudo       .-....G... .T........ .....A.... .......... .......... ....T...G. ..A....G.. .......... .......... ..........
P. coronopus B pseudo       .-....G... .T........ .....A.... .......... .......... ....T...G. ..A....G.. .......... .......... ..........
P. coronopus C pseudo       .-....G... .T........ .....A.... .......... .......... ....T...G. ..A....G.. .......... .......... ..........
P. coronopus D pseudo       .A........ .T........ .......... .......... .......A.. ........G. ..A....G.. .......... ..AG...... ..........
P. macrorhiza A pseudo      .-........ .T........ .......... .......... .......A.. ........G. ..A....G.. .......... ..A....... ..........
P. macrorhiza B pseudo      .C..G..... T......... .......... .......C.. .......A.. ........G. ..A....G.. .......... ..A....... ..........
P. subspathulata pseudo     .-....G... .T........ .....A.... .......... .......... ....T...G. ..A....G.. .......... .......... ..........
Jovellana                   .......... .......... .......... .......... .......... .......... ....C..G.. .......... .......... ..........
Streptocarpus               .......... .......... .......... .......... .......... .......... .A..C..G.. .......... .......... ..........
Mimulus                     .......... .......... .......... .......... .......... .......... ....C..G.. .......... .......... ..........
Aragoa                      .......... .......... .......... .......... A......... .......... ....C..G.. .......... .......... ..........
Digitalis                   .......... .......... .......... .......... .......... .......... ....C..G.. .......... .......... ..........
Globularia                  .......... .......... .......... .......... .......... .......... ....C..G.. .......... .......... ..........
P. lanceolata               .C..G..... ...A...... .......... .......... .......... ....C..... ....C..G.. C......... .......... ..........
P. sericea                  .C........ T......... .......... .......C.. .......... .......... .A..C..G.. C......... .......... ..........
P. crassifolia              .A....T... .A.A...... .......... .......C.. .......... ..A....... .A..T..C.. C......... .....TC..C .A...A.AA.
P. coronopus C funct        ......T... .A.A...... .CT....... .T.....C.. .......... ..A....... .A..T..C.. C......... .....TC.TC .T...A.AA.
P. macrorhiza B funct       ......T... .A.A...... .CT....... .T.....C.. .......... ..A....... .A..T..C.. C......... .....TC.TC .T...A.AA.
```

Additional File 1A - *atp1* alignment

```
                                  810        820        830        840        850        860        870        880        890        900
Nicotiana                 ACCACCAGGT CGTGAGGCTT TCCCAGGGGA TGTTTTCTAT ------TTAC ATTCCCGTCT CTTAGAAAGA GCGGCTAAAC GATCGGACCA GACAGGCGCA
Convolvulus               .......... .......... .......... ......T... ------.... .......... .......... .......... .......... ..........
Dinetus                   .......... .......... .......... ......T... ------.... .......... .......... .......... .......... ..........
C. europaea               .........C .......... .......C.. .......... ------.... .......... T......... ..C....... .......... ..........
P. coronopus A pseudo     ..T.T....C .......... ......AC.. .A......T. TTTTTT.... .....T.... T......... AGC....... .......... ..........
P. coronopus B pseudo     ....T....C .......... .......C.. .A......T. TTT---..TT .....T.... T......... AGC....... .......... ..........
P. coronopus C pseudo     ....T....C .......... .......C.. .A......T. TTT---..TT .....T.... T......... AGC....... .......... ..........
P. coronopus D pseudo     ....T....C .T.CG..... ..--.A.C.. .......... TT----.... .......... T......... A.C....... ..G....... ....A..A..
P. macrorhiza A pseudo    ....T....C .T.A...... .......C.. .......... TT----.... .......... T......... A.C....... .......... ....A.....
P. macrorhiza B pseudo    ....T....C .T.A...... .......C.. .......... TT----.... .......... T......... A.C....... .......... ....A.....
P. subspathulata pseudo   ....T....C .......... .......C.. .A......T. TTTTT-.... .....T.... T......... AGC....... .......... ..........
Jovellana                 .......... .......... .......A.. .......... ------.... .......... .......... ..C....... .......... ......T...
Streptocarpus             .......... .......... .......A.. .......... ------.... .......... .......... ..A....... .......... ..........
Mimulus                   .......... .......... .......A.. .......... ------.... .......... .......... .......... ....A..... ..........
Aragoa                    .......... .......... .......A.. .......... ------.... .......... .......... .......... .C........ ..........
Digitalis                 .......... .......... .......A.. .......... ------.... .......... .......... .......... .......... ..........
Globularia                .......... .......... .......A.. .......... ------.... .......... .......... .......... .......... ..........
P. lanceolata             ...T..G... .......... .......A.. .......... ------.... .......... A......... .....C.... ....C..... ..........
P. sericea                ...C...... ..G....... .......A.. .......... ------.... .......... .......... .....C.... ....A..... ..........
P. crassifolia            .......... .......... ....G..A.. .......... ------..G. .C..A..A.. .......... .....A...A ....A..... ...T......
P. coronopus C funct      ......G..C A.A....... ....G..A.. .......... ------..G. .C...A.A.. T......... ..C..G...A .........T C..G..A..T
P. macrorhiza B funct     ......G..C A.A....... ....G..A.. .......... ------..G. .C...A.A.. T......... ..C..G...A .........T C..G..A..T
```

```
                                  910        920        930        940        950        960        970        980        990       1000
Nicotiana                 GGTAGCTTGA CCGCCTTACC CGTCATTGAA ACACAGGCTG GAGACGTATC GGCCTATATT CCCACCAATG TGATCCCCAT TACTGATGGA CAAATCTGTT
Convolvulus               .......... .......... .......... .....A.... .......... .......... .......... .......... .......... ..........
Dinetus                   .......... .......... .......... .....A.... .......... .......... .......... .......... .......... ..........
C. europaea               .......... .......... .......... .....A.... .......... .......... .......... .......... .......... ..........
P. coronopus A pseudo     A......... ...T...... .......... .....A..G. .......... .A........ .......... .......... ...G...... ...T......
P. coronopus B pseudo     A......... ...T...... .......... .....A..G. .......... .A........ .......... .......... ...G...... ...T......
P. coronopus C pseudo     A......... ...T...... .......... .....A..G. .......... .A........ .......... .......... ...G...... ...T......
P. coronopus D pseudo     A......... .......... ....------ ----.A.... ......GC-- -------C.. .......... ........-. ...A.GG... ......CA..
P. macrorhiza A pseudo    A.....---- -----..... ....------ ----.A.... .......... .A........ .......... .......... ...A..G... ......CA..
P. macrorhiza B pseudo    A.....---- -----..... ....------ ----.A.... .......... .A........ .......... .......... ...A..GA.. ......CA..
P. subspathulata pseudo   A......... ..AT...... .......... .....A..G. .......... .A........ .......... .......... ...G...... ...T......
Jovellana                 .......... .......... .......... .....A.... .......... .......... .......... .....T.... .......... ..........
Streptocarpus             .....T.... .......... .......... .....A..C. ....T..T.. ...T...... ..T..T.... .C..TT.... .......... ..........
Mimulus                   .......... .......... .......... .....A..A. ....T..T.. ...T...... ..T..T.... .A..TT.... .......... ..........
Aragoa                    .......... .......... .......... .....A..A. ....T..T.. ...T...... ..T..T.... .A..TT.... .......... ..........
Digitalis                 .......... .......... .......... .....A..A. ....T..T.. ...T...... ..T..T.... .A..TT.... .......... ..........
Globularia                .......... .......... .......... .....A..A. ....T..T.. ...T...... ..T..T.... .A..TT.... .......... ..........
P. lanceolata             ..A.....A. .......... G..T...... ..T..A..A. .G........ A......... ..A....... .C...T.... ...A...... ..........
P. sericea                ..A.....A. .G........ T......... .....A..A. .G........ .......... ..A....... .C...T.... ...A...... ..........
P. crassifolia            ..A.....A. ....A..... ......C... .....A..G. .......T.. C..T...... ..A..T.... .C...T.... ...A...... ..G..A..C.
P. coronopus C funct      ..A.....A. ....A..... .......... .....A..G. .......T.. C..T...... ..T..G.... .C...T.... ...G...... ..G..A..C.
P. macrorhiza B funct     ..A.....A. ....A..... .......... .....A..G. .......T.. C..T...... ..T..G.... .C...T.... ...G...... ..G..A..C.
```

```
                                 1010       1020       1030       1040       1050       1060       1070       1080       1090       1100
Nicotiana                 TGGAAACAGA GCTCTTTTAT CGCGGAATTA GACCTGCGAT TAACGTCGGC TTATCTGTCA GTCGCGTCGG GTCTGCCGCT CAGTTGAAAA CTATGAAACA
Convolvulus               .......... .......... .......... .......T.. .......... .......... .......... .......... .......... ..........
Dinetus                   .......... .......... .......... .......T.. .......... .......... .......... .......... .........G ..........
C. europaea               .......... ...A...... .......... .......T.. .......... ........A. .......... .......... .........G ..........
P. coronopus A pseudo     .....C.G.. .......... T...A..... .......T.. ....A..... ........A. ...A...... .......... .........G .G.G......
P. coronopus B pseudo     .....C.G.. .......... T...A..... .......T.. ....A..... ........A. ...A...... .......... .........G ...G......
P. coronopus C pseudo     .....C.G.. .......... T...A..... .......T.. ....A..... ........A. ...A...... .......... .........G ...G......
P. coronopus D pseudo     .......... ...T...... .......... ..T....T.. ..G.A.T... ....T...A. A...T..... ....A..... .........G ...G......
P. macrorhiza A pseudo    .......... .......... .......... ..T....T.. ..G.ACT... ........A. A......A.. ....C....G ....A....G ...G......
P. macrorhiza B pseudo    .......... .......... .......... ..T....T.. ..G.ACT... ........A. A......A.. ....C....G ....A....G ...G......
P. subspathulata pseudo   .....C.G.. .......... T...A..... .......T.. ....A..... ........A. ...A...... .......... .........G ...G......
Jovellana                 .......... .......... .........C .......T.. .........A .......... .......... .......... .........G ..........
Streptocarpus             .......... .......... .......... .......T.. ...T.....A .......... .......... .......... .........G ..........
Mimulus                   .......... .......... .......... .......T.. ......A..A .......... .......... .......... .........G ..........
Aragoa                    .......... .......... .......... .......... .........A .......... .......... .......... .......... ..........
Digitalis                 .......... .......... .......... .......... .........A .......... .......... .......... .......... ..........
Globularia                .......... .......... .........C .......T.. .........A .......... .......T.. .......... .........G ..........
P. lanceolata             .......... .........C .........C .......T.. .........A .....C..T. .......... .......... ..A....... .G........
P. sericea                .......... .........C ..T......C .......T.. .........A .....C.... .......... ...G...... ..A..A.... .........G
P. crassifolia            .......... A........C A.A......C ....G..C.. ......G..A .....C.... ....A..A.. A..C..A..A .......... .C......A.
P. coronopus C funct      .......C.. ...T.....C A.A......C ....C..T.. .........A .....C.... ....A..G.. A..C.....A .......... .C........
P. macrorhiza B funct     .......C.. ...T.....C A.A......C ....C..T.. .........A .....C.... ....A..G.. A..C.....A .......... .C........
```

```
                                 1110       1120       1130       1140       1150       1160       1170       1180       1190       1200
Nicotiana                 AGTCTGCGGT AGTTCAAAAC TGGAATTGGC ACAATATCGC GAAGTGGCCG CCCTTGCTCA ATTTGGCTCA GACCTTGATG CTGCGACTCA GGCATTACTC
Convolvulus               .......... ....T..... .......... .......... .......... ..T....... .......... .......... .A........ ..........
Dinetus                   .......... ....T..... .......... .......... .......... ..T....... .......... .......... .A........ ..........
C. europaea               .......... ....T..... .......... .......... .......... ..T....... .......... .......... .A........ ..........
P. coronopus A pseudo     ...A...... ....T....T ...C...... ........A. ...A..AT.. ..T....... .A........ .......... .A..
P. coronopus B pseudo     ...A...A.. ....T....T ...C...... ........A. ...A..AT.. ..T....... .A........ .......... .A...G.... A......T..
P. coronopus C pseudo     ...A...A.. ....T....T ...C...... ........A. ...A..AT.. ..T....... .A........ .......... .A...G.... A....A
P. coronopus D pseudo     ...A..T... ....T....T .A.C...... ........A. ...A..A... ..T....... .A........ .......... .A..A..... ..T.......
P. macrorhiza A pseudo    ...A..T... ....T....T ...C...... ........A. ...A..A... ..T.....T. .A........ .......... .A..A..... ..T.......
P. macrorhiza B pseudo    ...A..T... ....T....T ...C...... ........A. ...A..A... ..T.....T. .A........ .......... .A..A..... ..T.......
P. subspathulata pseudo   ...A...... ....T....T ...C...... ........A. ...A..AT.. ..T....... .AA....... .......... .A...G.... .......T..
Jovellana                 ...A.....G ....T..... .A........ .........A .....A.... ..T....... .......... .......... ....C..... ..........
Streptocarpus             ...A.....G ....T..... .A........ .........A .....T.... ..T....... .......... .......... ....C..... ..........
Mimulus                   ...A.....G ....T..... .A........ .......A.A .....A.... ..T....... .......... .......... ....C..... ..........
Aragoa                    ...A.....G ....T..... .C........ .........A .....A.... ..T....... .......... .......... ....C..... A.........
Digitalis                 ...A.....G ....T..... .A........ .........A .....A.... ..T....... .......... .......... ....C..... ..........
Globularia                ...A.....G ....T..... .A........ .........A .....T.... ..T....... .......... ..T....... ....C..... ..........
P. lanceolata             ...T...... ....T..... .......... .......A.A .....A.... ..T....C.. .........C ..T..C.... ....C..... ..........
P. sericea                ...G.....C ....T..... .C........ .......A.A .....A.... ..T....C.. .......... ..T..G.... ....C..... ..........
P. crassifolia            ...A.....A ....T..... .C........ .......A.A .....C..G. ..T....A.. .......... .....A.... .G..C..... ..........
P. coronopus C funct      ...T...... ....T..... .C........ .......A.A .....C..A. ..T....G.. ......G..T .....A.... .G..C..... ...
P. macrorhiza B funct     ...T...... ....T..... .C........ .......A.A .....C..A. ..T....G.. ......G..T .....A.... .G..C..... ...
```

Additional File 1A - *atp1* alignment

```
                                  1210       1220       1230       1240       1250       1260       1270       1280       1290       1300
                         ....|....| ....|....| ....|....| ....|....| ....|....| ....|....| ....|....| ....|....| ....|....| ....|....|
Nicotiana                AATAGAGGTG CAAGGCTGAC AGAAGTACCG AAACAACCAC AATATGCACC ACTGCCAATT GAAAAA---- --CAAATTCT AGTCATTTAT GCAGCTGTCA
Convolvulus              .......... .......T.. ........T. .......... .......... .......... ......---- --........ ...A...... ..........
Dinetus                  .......... .......... ........T. .......... .......... .......... ......---- --........ ...A...... ..........
C. europaea              .......... .......... ........T. .......... .......... .......... ......---- --......A. ...A...... ..........
P. coronopus A pseudo
P. coronopus B pseudo    .......... .......... ........T. ......TT.. .......... .T....C... ......AA-- --......A. ...A...... ......A...
P. coronopus C pseudo
P. coronopus D pseudo    .......... .......... .......TT. .......... .......... .TG....... ......AA-- --......A. ...A..
P. macrorhiza A pseudo   .......... .......... .......TT. .........T .......... .TG....... ......AAA- --......A. ...A...... ..C.......
P. macrorhiza B pseudo   .......... .......... .......TT. .........T .......... .TG....... ......AA-- --......A. ...A..
P. subspathulata pseudo  .......... .......... ........T. ......TT.. .......... .T....C... ......AAAA xx......A. ...A...... ......A...
Jovellana                ........G. .......... ....A...TC .......... .....A.... .T........ ......---- --......A. ...T...... ..T.....G.
Streptocarpus            ........G. .T..A..T.. ....A...TC .......... .....A.... .T........ ......---- --........ ........T. ..T.......
Mimulus                  ........G. .......... ....A...TC .......... .....A.... .T........ ......---- --........ .........C .....G....
Aragoa                   ........G. .......... ....A...TC .......... .....A.... .T........ ......---- --G....... .........C .....G....
Digitalis                ........G. .......T.. ....A...TC .......... .....A.... .T........ ......---- --G....... .........C .....G....
Globularia               ........G. .......... ....A...TC .......... .....A.... .T........ ......---- --........ ....
P. lanceolata            ........G. .....T.A.. ....A...T. .......... .......... .T.A..C... ......---- --G....... ...T.....C ..........
P. sericea               ........A. .....T.... ....A...TC ....G.G... .......... .T.A..C.
P. crassifolia           ........A. .C...T.... ....A...TC .......... .....AAT.. .T.AG.C... ......---- --G....... .........C ..T.......
P. coronopus C funct
P. macrorhiza B funct


                         ....|
Nicotiana                ATGGA
Convolvulus              .....
Dinetus                  .....
C. europaea              .....
P. coronopus A pseudo
P. coronopus B pseudo    .....
P. coronopus C pseudo
P. coronopus D pseudo
P. macrorhiza A pseudo   .....
P. macrorhiza B pseudo
P. subspathulata pseudo  .....
Jovellana                .....
Streptocarpus            .....
Mimulus                  .....
Aragoa                   .....
Digitalis                .....
Globularia
P. lanceolata            .....
P. sericea
P. crassifolia           .....
P. coronopus C funct
P. macrorhiza B funct
```

Additional File 1B - *atp6* alignment

```
                               10         20         30         40         50         60         70         80         90        100
                       ....|....| ....|....| ....|....| ....|....| ....|....| ....|....| ....|....| ....|....| ....|....| ....|....|
Nicotiana              GTCCTACTTT TGGTTTATTT TGTTACTAAA AAGGGAGGAG GAAACTCAGT ACCAAATGCT TGGCAATCCT TGGTAGAGCT TATTTATGAT TTCGTGCTGA
Convolvulus            .........C ..C....... .......... .......... .......... .......... .......... .......... .......... .......C..
Dinetus                .........C ..C....... .......... .......... .......... .......... .......... .......... .......... .......C..
C. europaea            .........C ..C....... .......... .......... .......... .......... .......... .......... .......... .......C..
P. coronopus C pseudo  .........C ..C....... .......... ..A......A ...T...... ........T. .......... .......... .......... ...T.TTC..
P. coronopus D pseudo  A........C ..C....... .......... ......A... ...C...... ........T. .......... .......... ........C. ..TA...C..
P. macrorhiza A pseudo A........C ..C....... .......... ......A... ...C...... ........T. ...T...... .......... ........C. ..T....C..
P. macrorhiza B pseudo ..G.C....C ..C....... .......... .......... ...C...... ........T. .......... .......... A.......C. ..T....C..
Jovellana              .........C ..C....... .......... .......... .......... .......... .......... .......... .......... ..........
Streptocarpus          .......... ..T..C.... ......A... .......... ....G.T... C......... .......... .......... .C........ ..........
Mimulus                .......... .T...C.... .......... .......... .......... .......... .......... .......... .......... .......C..
Aragoa                 .......... .T...C.... .......... .......... .......... .......... .......... .......... .......... .......C..
Digitalis              .......... .T..GC.... ...G...... .......... .......... .......... .......... .......... .......... .......C..
Globularia             .......... .T...C.... .......... .......... ......T... .......... .......... .......... .C........ ..........
P. lanceolata          .......... .TC.GC.... ...G..G... ..C....... ....T.T... G..C...... .......... .......... .......... .....CTC..
P. crassifolia                       .C..... ...G..CC.. ..T....... .G..A.T... G..C...... .........G .A........ .......... ......TC..
P. coronopus C funct                 .C..... ...C..AC.. ..T..G.... .G..A.T... C..C...... .........G .T.....A.. .......... .....CTC..
P. macrorhiza B funct                .C..... ...C..AC.. ..T..G.... .G..A.T... C..C...... .........G .T.....A.. .......... .....CTC..
```

```
                              110        120        130        140        150        160        170        180        190        200
                       ....|....| ....|....| ....|....| ....|....| ....|....| ....|....| ....|....| ....|....| ....|....| ....|....|
Nicotiana              ACCCGGTAAA CGAACAA--- ---ATAGGTG GTCTTTCCGG AAATGTTAAA CAAAAGTTTT CCCCTCGCAT CTCGGTCACT TTTACTTTTT CGTTATTTTG
Convolvulus            .......... .......--- ---....... .......... .......... .......... .....T.... .......... .......... ........C.
Dinetus                .......... .......--- ---....... .......... .......... .......... .....T.... .......... .......... ........C.
C. europaea            .......... .......--- ---....... .......... .......... .......... .....T.... .......... .......... ........C.
P. coronopus C pseudo  ...T...... .......--- ---....... ......T... .......... .......... .T...T.... .......... .......... ........C.
P. coronopus D pseudo  .......... .A.....ACA Axx..G.... .......... .......... .......... .T...T.... ......T--C .......... ........CA
P. macrorhiza A pseudo .......... .......--- ---..G.... .......... .......... .......... .T...T.... ......T--C .......... ........CA
P. macrorhiza B pseudo .......... .......--- ---....... .......... .......... .......... .T...T.... .......--C .......... ........CA
Jovellana              ...T...... .......--- ---....... .......... ......G... .......... T....T.... ......T... .......... ..........
Streptocarpus          ...T...... .......--- ---....... .......... ......G... .......A.. T....T.... ......T... .......... ........C.
Mimulus                ...T...... .......--- ---....... .......... ......G... .......... T....T.... ......T... .......... ........C.
Aragoa                 ...T...... .......--- ---....... .......... ......G... .......... T....T.... ......T... .......... ........C.
Digitalis              ...T...... .......--- ---....... .......... ......G... .......... T....T.... ......T... .......... ........C.
Globularia             ...T...... .......--- ---....... .......... ......G... .......... T....T.... ..T...T... .......... T.........
P. lanceolata          ...T...... .......--- ---.....C. .......... ......G... .......... T....T.... ..T...T... ...CT..... T...C....C
P. crassifolia         .T.TC..... .......--- ---..T..A. .G..G..... ......C... .......... T....GT... ..TA..T... .......... TA......C.
P. coronopus C funct   .T.TA..C.. .......--- ---..C..A. .CT.G..... ......C... .......... T....GT... ..T...G... ........C. T.......A.
P. macrorhiza B funct  .T.TA..C.. .......--- ---..C..A. .CT.G..... ......C... .......... T....GT... ..T...G... ........C. T.......A.
```

```
                              210        220        230        240        250        260        270        280        290        300
                       ....|....| ....|....| ....|....| ....|....| ....|....| ....|....| ....|....| ....|....| ....|....| ....|....|
Nicotiana              TAATCCCCAG GGTATGATAC CTTATAGCTT CACAGTTACA AGTCAT---- --TTTCTCAT TACTTTGGGT CTCTCATTTT CTATTTTTAT TGGCATTACT
Convolvulus            .......... .......... .......... .......... ......---- --........ .......... .......... .G........ C.........
Dinetus                .......... .......... .......... .......... ......---- --........ .......... .......... .G........ ..........
C. europaea            .......... .......... .......... ......G... ......---- --........ .......... .......... ..C....... ..........
P. coronopus C pseudo  A.......C. ......C... .......T.. ......G... ...T..---- --...T.... .......... ....T....- --T.....-- --........
P. coronopus D pseudo  ....T..... .......... .......... ......G... ...T..TTAT xx........ .......A.. T...T..... -.C....... ..........
P. macrorhiza A pseudo ....T..... .......... .......... ......G... ...T..TTAT xx........ .......A.. T...T..... TC-....... ..........
P. macrorhiza B pseudo .......... .A........ .......... T.....G... ...T..---- --........ ......A... T...T..... ..T....... ..........
Jovellana              .......... .......... .......... ......G... ......---- --........ .......... .......... ..C....... ..........
Streptocarpus          .....T.... .......... .......... .......... ......---- --........ .......... .......... ..C....... ..........
Mimulus                .....TT... .......... .......... .......... ......---- --........ .......... .......... ..C....... ..........
Aragoa                 .....T.... .......... .......... ......G... ......---- --........ .......... .......... ..C....... ..........
Digitalis              .....T.... .......... .......... .......... ......---- --........ .......... .......... ..C....... ..........
Globularia             .....TT... .......... .......... .......... ......---- --........ .......... .......... ..C....... ..........
P. lanceolata          .....TA..A .......... .......T.. ......G... ......---- --........ .......... .......... ..C.....C. ...T......
P. crassifolia         .....TA... ..A.....C. .C.....T.. ......C... ......---- --.....A.. C.....A..A ..G..C.... .CC.A...C. G..G......
P. coronopus C funct   .....TA..A ..A....... .C.....T.. ......C... ......---- --.....T.. C.....A..A A.G..C.... .CC.....C. A..G.....C
P. macrorhiza B funct  .....TA..A ..A....... .C.....T.. ......C... ......---- --.....T.. C.....A..A A.G..C.... .CC.....C. A..G.....C
```

```
                              310        320        330        340        350        360        370        380        390        400
                       ....|....| ....|....| ....|....| ....|....| ....|....| ....|....| ....|....| ....|....| ....|....| ....|....|
Nicotiana              ATAGTGGGAT TTCAAAAAAA TGGGCTTCAT TTTTTA---A GCTTCTTATT ACCTGCAGGA GTCCCACTGC CATTAGCACC TTTTTTAGTA CTCCTTGAGC
Convolvulus            .......... ......G... .......... .....T---. .......... .......... .......... .G........ .......... ..........
Dinetus                .......... ......T... .......... .....T---. .......... .......... .......... .G........ .......... ..........
C. europaea            .......... ......G... .......... .....T---. .......... .......... .......... .G........ .......... ..........
P. coronopus C pseudo  .G........ ......G... .......... .....T---. .......... .......C.. ....T.T... .G......T. ...C...... ..........
P. coronopus D pseudo  .G....A... ......G... ....T..... .....T---. .......... .......... ...T....A. .G........ ...C..G... ........A.
P. macrorhiza A pseudo .G....AA.. ......G... ....T..... .....T---. .......... .......... ........A. .G........ ...C..G... ........A.
P. macrorhiza B pseudo .G........ ......G... ....T..... .....TTxx. .......... .......... ........A. .G....T... ......G... ..........
Jovellana              .......... .......... .......... ......---. ......C... ...C...... .......... .G........ .......... ..........
Streptocarpus          .......... ......G... .......... ......---. ......C... ...C...... .......... .G........ .......... ..........
Mimulus                .......... .......... .......... ......---. ......C... ...C...... .......... .G........ .......... ..A.......
Aragoa                 .......... .......... .......... ......---. ......CC.. ...C...... .......... .G........ .......... ..........
Digitalis              .......... .......... .......... ......---. ......CC.. ...C...... .......... .G........ .......... ..........
Globularia             .......... .......... .........C ......---. .......C.. ...C...... .......... .G........ .......... ..........
P. lanceolata          .......... ........C. .......... ......---. ......CC.. ...C...... .......... .C........ C......... ..........
P. crassifolia         .....C.CT. .......GC. C..A...... ......---. ......CC.. ...G..T..G .......... .C........ C......... ..........
P. coronopus C funct   ..T..C.CT. .......GC. C..A..A..C ..C...---. .......C.. ...C..C..C ..A..T..A. .C......G. C......... ..A.....A.
P. macrorhiza B funct  ..T..C.CT. .......GC. C..A..A..C ..C...---. .......C.. ...C..C..C ..A..T..A. .C......T. C......... ..A.....A.
```

Additional File 1B - *atp6* alignment

```
                                  410        420        430        440        450        460        470        480        490        500
                             ....|....| ....|....| ....|....| ....|....| ....|....| ....|....| ....|....| ....|....| ....|....| ....|....|
Nicotiana                    TAATCCCTTA TTGTTTTCGA GCATTAAGCT CAGGAATACG TTTATTTGCT ---------- --------AA TATGATGGCC GGTCATAGTT CAGTAAAGAT
Convolvulus                  ........C. .........C .......... .......... .......... ---------- --------.. .......... .......... ..........
Dinetus                      ........C. .........C .......... .......... .......... ---------- --------.. .......... .......... ..........
C. europaea                  ........C. .........C .......... .......... .......... ---------- --------.. .......... .......... ..........
P. coronopus C pseudo        C.......C. .........C .......... .......... .......A.. ---------- --------.. ......A... .-........ ....T.....
P. coronopus D pseudo        ........C. C......A.C .......... .........T .......A.. ATTTTATTTA CTAATATx.. ...A...... .-........ ....C.....
P. macrorhiza A pseudo       ........C. C......A.C .......... .........T .......A.. ---------- --------.. ...A...... .-........ ....C.....
P. macrorhiza B pseudo       ........C. .........C .......... .......... .......A.. ---------- --------.. ...A...... .-........ ....C.....
Jovellana                    ........C. .........C .......... T......... .......... ---------- --------.. .......... .......... ..........
Streptocarpus                ....TT.... .........C .......... T......... .......... ---------- --------.. .......... .......... T.........
Mimulus                      ........C. .........C .......... T......... .......... ---------- --------.. .......... .......... ..........
Aragoa                       ........C. .........C .......... T......... .......... ---------- --------.. .......... .......... ..........
Digitalis                    ........C. .........C .......... T......... .......... ---------- --------.. .......... .......... ..........
Globularia                   .....T.... .........C .......... T......... .......... ---------- --------.. .......... .......... T.........
P. lanceolata                ........C. .........C ........T. T......... C......... ---------- --------.. .........T .......... T...C.....
P. crassifolia               .....T.... ..CC.....C ........T. T......... A......... ---------- --------.. .......... ..A....... T...C.....
P. coronopus C funct         .T...T.... C.CC.....C ..T.....T. T......... A......... ---------- --------.. .......... ..A....... T...C.....
P. macrorhiza B funct        .T...T.... C.CC.....C ..T.....T. T......... A......... ---------- --------.. .......... ..A....... T...C.....

                                  510        520        530        540        550        560        570        580        590        600
                             ....|....| ....|....| ....|....| ....|....| ....|....| ....|....| ....|....| ....|....| ....|....| ....|....|
Nicotiana                    TTTAAGTGGG TTCGCTTGGA CTATGCTATG TATGAATGAT CTTTTATATT TCATAGGGGA TCTTGGTCCT TTATTTATAG TTCTTGCATT AACCGGTCTG
Convolvulus                  .......... .......... .......... .......... .......... .......A.. .......... .......... ....C..... .........T
Dinetus                      .......... .......... .......... .......... .......... .......A.. .......... .......... .......... .........T
C. europaea                  .......... .......... .......... .......... .....C.... .......A.. .......... .......... .......... .........T
P. coronopus C pseudo        .......A.. ..A....... T......... .......... .....C.... .......A.. .......... .......... .......... ........CT
P. coronopus D pseudo        .......A.A .......... T......... .......... -....C.... .......A.. .......... .......... .......... ..T......T
P. macrorhiza A pseudo       .......A.A .......... T......... .......... -....C.... .......A.. .......--- ------.... .......... ..T......T
P. macrorhiza B pseudo       .......A.A .......... T......C.. .......... -....C.... .......AA. .......... .......G.. .......... ..T......T
Jovellana                    .......... .C........ .......... .......... .....C.... .......... ..C....... .......... .......... ..........
Streptocarpus                .......... .......... .......... .......... .....C.... .......... .......... .......... .......... .........T
Mimulus                      .......... .C........ .......... .......... .....C.... .......... ..C....... .......... .......... ..........
Aragoa                       .......... .C........ .......... .......... .....C.... .......... ..C....... .......... .......... ..........
Digitalis                    .......... .C........ .......... .......... ..G..C.... .......... ..C....... .......... .......... ..........
Globularia                   .......... .......... .......... .......... .....C.... .......A.. .......... .......... .......... ..........
P. lanceolata                .........C .......... .......... .......... .....T.... .......TC. .......... ........C. ....G..... .........C
P. crassifolia               .........A .......... .C........ .......... ..AG.C.... .......AC. ...A..A..A .....G..C. ....C..... ...G..C..C
P. coronopus C funct         .........A .......... .C.....T.. .......... ...G.C.T.. ....T..AC. ...A..A..C ...GCC..C. ....C..G.. ...T..A..C
P. macrorhiza B funct        .........A .......... .C.....T.. .......... ...G.C.T.. ....T..AC. ...A..A..C ...GCC..C. ....C..G.. ...T..A..C

                                  610        620        630        640        650
                             ....|....| ....|....| ....|....| ....|....| ....|....| .
Nicotiana                    GAATTAGGTG TAGCTATATC ACAAGCTCAT GTTTCTACGA TC---TTAAT C
Convolvulus                  .......... .......... .......... .......... ..---.C... .
Dinetus                      .......... .......... .......... .......... ..---.C... .
C. europaea                  .......... .......... .......T.. .......... ..---.C... .
P. coronopus C pseudo        .......... .......... .......T.. .......... ..---.C.
P. coronopus D pseudo        A......... .......... .......T.. .......... C.TCx.C.
P. macrorhiza A pseudo       A......... .......... .......T.. .......... C.---.C... .
P. macrorhiza B pseudo       A......... .......... .......T.. .......... C.---.C.
Jovellana                    .......... .......... .......... .......... ..---.C... .
Streptocarpus                .......... .......... .......... .......... ..---.C... .
Mimulus                      .......... .......... .......... .......... ..---.C... .
Aragoa                       .......... .......... .......... .......... ..---.C... .
Digitalis                    .......... .......... .......... .......... ..---.C... .
Globularia                   .......... .........T .......T.. ....T..... ..---..... .
P. lanceolata                .......... .........T .......... ....T..... ..---.C... .
P. crassifolia               .......... .C.......T .......... ....T..C.. ..---.C.
P. coronopus C funct         ..G....... .C..C..T.T .........C ....T..C.. ..---.C.
P. macrorhiza B funct        ..G....... .C..C..T.T .........C ....T..C.. ..---.C.
```

Additional File 1C - *matR* alignment

```
                                  10         20         30         40         50         60         70         80         90        100
                         ....|....| ....|....| ....|....| ....|....| ....|....| ....|....| ....|....| ....|....| ....|....| ....|....|

Nicotiana                CATATAACTG CTTGCCACCT ACGCTCCGCC ---ATCCATT CAAAGTTTAG GAACCTAGGT ---------A ATAGTATCCC GATCAAAGAG CTGACGAAGG
Convolvulus              .......... .......... .......... ---....... .C........ ......C... ---------. ........T. .......C.. ..........
Dinetus                  .......... .......... .......... ---....... .C........ ......C... ---------. ........T. .......C.. ..........
C. europaea              .......... .......... .......... ---....... .C........ ......C... ---------. ........T. .......C.. ..........
P. coronopus C pseudo      A....... .C........ ....G..... Cxx....... .C......G. ......C... ---------. .-.A....T. ..G....C.. ..A...C...
P. coronopus D pseudo      ....G... ...A.T.... .T.TG..A.. ---....... .C........ A.......T. AGGTTATACT ........T. A......C.A .....A...A
P. macrorhiza A pseudo     ....G... .....T..-. ...T.T.A.. ---....... .C........ A......... AGGT-----. ........T. A.....GC.. .....A....
P. macrorhiza B pseudo     ....G... .....T.... ...T.T.A.. ---....... .C........ A......... AGGT-----. ........T. A.....GC.. .....A....
P. subspathulata pseudo    ....G... ...A.T.... .T.TG..A.. ---....... .C........ A......... AGGTTATACT ........T. A......C.. .....A....
Jovellana                .......... .......... ......G... ---....... .C........ .......... ---------. .......... .......C.. ..........
Streptocarpus            .......... .......... ......G... ---....... .G........ .......C.. ---------. .......... .......C.. ..........
Mimulus                  .......... .......... ......G... ---....... .C........ .......... ---------. .......... .......C.. ..........
Aragoa                   .......... .......... ......G... ---....... .C........ .......... ---------. .......... .......C.. T.........
Digitalis                .......... .......... ......G... ---....... .C........ .......... ---------. .......... .......C.. ..........
Globularia               .......... ....G..... ......G... ---....... .C........ .......... ---------. .......... .......C.. ..........
P. lanceolata            ....G..... .......... ...A..G.TT ---....... .C..A.C... ........AG ---------. .......... A......... T.........
P. crassifolia           ..ACG...G. .AG....T.. ...AG.G... ---.....GG ..C.CC.... .........A ---------. .A.CC..GA. CCAA...... .G..A.....
P. coronopus C funct     ..ACG...C. .AG.A.T... CA.AG.G... ---...A.AG A.C.CC.A.A C........A ---------. .A.CC.AA.A C.CGC....A AG..AC....
P. macrorhiza B funct    ..ACG...C. .AG.A.T... CA.AG.G... ---...A.AG A.C.CC.A.A C........A ---------. .A.CC.AA.A C.CGC....A AG..AC....

                                 110        120        130        140        150        160        170        180        190        200
                         ....|....| ....|....| ....|....| ....|....| ....|....| ....|....| ....|....| ....|....| ....|....| ....|....|

Nicotiana                GGATGAGCGG AACAGGGAGT CTACTGGACG CGGTTCAACT AGCGGAGACT CTTGGAACAG CTGGAGTAAG AAGTCCCCAA GTGAGCGTCT TATGGGGGGC
Convolvulus              .......... ....TTCC.. .......... .......... .......... .......... .....A.... .......... ..T.....A. ........A.
Dinetus                  .......... ....TTCC.. .......... .......... .......... .......... .....A.... .......... ..T.....A. ........A.
C. europaea              .......... ....TTCC.. .......... .......... .......... .......... .....A.... .......... ..T.....A. ........A.
P. coronopus C pseudo    .......... ....TTCC.. A.......TA .......G.. ........T. T........A .....A.... ....T----- ---.....A. .G......A.
P. coronopus D pseudo    .....GT... ....TTCC.. ......A... .......... .......... T.C......A .....A.... ....TTA... T.T.....A. ........A.
P. macrorhiza A pseudo   ......T... ....TTCC.. ......A... T......... T.T....... T........A .....A.... ....TT.... ..T.....A. ........A.
P. macrorhiza B pseudo   ......T... ....TTTC.. ......A... .......... T.T....... T........A .....A.... ....T..... ..T.....A. ........A.
P. subspathulata pseudo  ......T... ....TTCC.. ......A... .......... .......... T.C......A .....A.... ....TT.... T.T.A...A. ........A.
Jovellana                .......... .......... .......... .......... .......... .......... .......... .......... ........A. ........A.
Streptocarpus            .......... .......... .......... .......... .......... .......... .......... .G........ ........A. ........A.
Mimulus                  .......T.. .......... .......... .......... .......... .......... .......... .......... .....T..A. ........A.
Aragoa                   .......... .......... .......... .......... .......... .......... .......... .......... ........A. .......AA.
Digitalis                .......... .......... .......... .......... .......... .......... .......... .......... ........A. ........A.
Globularia               .......... .......... .......... .......... .......... .......... .......... .......... ........A. ........A.
P. lanceolata            .CC.TTA... ..G..AC... .C...A.... .......... G.....A... .......... .......T.T .....T.G.. A.....T.A. ........A.
P. crassifolia           CC------AT C.GCCCA... ....GA..A. .CC...C... ...A..A... A.G....G.. .C.....C.. .....TA.C. A.C...C.AC ..G...CCA.
P. coronopus C funct     CA------.. C.G.ACA... T.CACA..A. .T------A. ....C.A..C A.CCA..G.. AA.....C.T .....TA.C. A.CC.T---- -----.CCT.
P. macrorhiza B funct    CA------.. C.G.ACA... T.CAAA..A. .T------A. ....C.A..C A.CCA..G.. AA.....C.T .....TA.C. A.CC.T---- -----.CCT.

                                 210        220        230        240        250        260        270        280        290        300
                         ....|....| ....|....| ....|....| ....|....| ....|....| ....|....| ....|....| ....|....| ....|....| ....|....|

Nicotiana                CGTCAAGCAC ATACGGCAAG GATCAAGGGA GATC------ ---------- ---------- ---------- ---------- ---------- ----------
Convolvulus              .......... ..C....... .........G ...T------ ---------- ---------- ---------- ---------- ---------- ----------
Dinetus                  .......... ..C....... .........G ...T------ ---------- ---------- ---------- ---------- ---------- ----------
C. europaea              .......... ..C....... .........G ...T------ ---------- ---------- ---------- ---------- ---------- ----------
P. coronopus C pseudo    ..C.....G. ..C......A ...T.....G ...T------ ---------- ---------- ---------- ---------- ---------- ----------
P. coronopus D pseudo    .C.T...... ..C....... .......A.T ...T------ ---------- ---------- ---------- ---------- ---------- ----------
P. macrorhiza A pseudo   .C........ ..C....... .........T ...T------ ---------- ---------- ---------- ---------- ---------- ----------
P. macrorhiza B pseudo   .C........ ..C....... .........T ...T------ ---------- ---------- ---------- ---------- ---------- ----------
P. subspathulata pseudo  .T.T...... ..C....... .......--- ---------- ---------- ---------- ---------- ---------- ---------- ----------
Jovellana                .......... ..C....... .........G ....------ ---------- ---------- ---------- ---------- ---------- ----------
Streptocarpus            .......... ..C....... .........G ....------ ---------- ---------- ---------- ---------- ---------- ----------
Mimulus                  .......... ..C....... .G.......G ....------ ---------- ---------- ---------- ---------- ---------- ----------
Aragoa                   ...A...... ..C....... ........TT ....------ ---------- ---------- ---------- ---------- ---------- ----------
Digitalis                ...A...... ..C....... .........G ....------ ---------- ---------- ---------- ---------- ---------- ----------
Globularia               .......... ..C....... .........G ....------ ---------- ---------- ---------- ---------- ---------- ----------
P. lanceolata            .A........ ..C..C...A ......AAA. A..A------ ---------- ---------- ---------- ---------- ---------- ----------
P. crassifolia           .A........ .G...A...A ..AG..AA.. C...TTCCAG AGTCCGGGGA CCAGAAAAGG AGAAAAACCA GAGCCATCCA AGAATCCACC CAAAAGGTTC
P. coronopus C funct     .T.A..C..A .....AA.CA A.AG..CA.. AGG.TTACAG AGTCCGGAGA ACCAAAAAGA AGAACAAAGA GAGACAGACA AAAAGACAGA GAAAAGGGGC
P. macrorhiza B funct    .T.A..C... .....AA.CA A.AG..CA.. AGG.TTACAG AGTCCGGAGA ACCAAAAAGA AGAACAAAGA GAGACAGACA AAAAGACAGA GAAAAGGGGC

                                 310        320        330        340        350        360        370        380        390        400
                         ....|....| ....|....| ....|....| ....|....| ....|....| ....|....| ....|....| ....|....| ....|....| ....|....|

Nicotiana                ---------- ---------- ---------- ---------- ---------- ---------- ---------- ---------- ---------- ----------
Convolvulus              ---------- ---------- ---------- ---------- ---------- ---------- ---------- ---------- ---------- ----------
Dinetus                  ---------- ---------- ---------- ---------- ---------- ---------- ---------- ---------- ---------- ----------
C. europaea              ---------- ---------- ---------- ---------- ---------- ---------- ---------- ---------- ---------- ----------
P. coronopus C pseudo    ---------- ---------- ---------- ---------- ---------- ---------- ---------- ---------- ---------- ----------
P. coronopus D pseudo    ---------- ---------- ---------- ---------- ---------- ---------- ---------- ---------- ---------- ----------
P. macrorhiza A pseudo   ---------- ---------- ---------- ---------- ---------- ---------- ---------- ---------- ---------- ----------
P. macrorhiza B pseudo   ---------- ---------- ---------- ---------- ---------- ---------- ---------- ---------- ---------- ----------
P. subspathulata pseudo  ---------- ---------- ---------- ---------- ---------- ---------- ---------- ---------- ---------- ----------
Jovellana                ---------- ---------- ---------- ---------- ---------- ---------- ---------- ---------- ---------- ----------
Streptocarpus            ---------- ---------- ---------- ---------- ---------- ---------- ---------- ---------- ---------- ----------
Mimulus                  ---------- ---------- ---------- ---------- ---------- ---------- ---------- ---------- ---------- ----------
Aragoa                   ---------- ---------- ---------- ---------- ---------- ---------- ---------- ---------- ---------- ----------
Digitalis                ---------- ---------- ---------- ---------- ---------- ---------- ---------- ---------- ---------- ----------
Globularia               ---------- ---------- ---------- ---------- ---------- ---------- ---------- ---------- ---------- ----------
P. lanceolata            ---------- ---------- ---------- ---------- ---------- ---------- ---------- ---------- ---------- ----------
P. crassifolia           TATACTCCAG CAGGAAGAAG AGCCCAAGAA GAAAGAGACA GAAGAAAGGA AAAGGAAGAA TCAGAGAAAG AACGAGAAAT CGAAGGGAAA AGAGCAGAAA
P. coronopus C funct     CATACCACAA CAGAAAAAAG AAAACAAGAA GAAAGAGAAA GAAGAAACCC AGAAAAAAGA GAAAGAAGAA AACAAGAAGA AAGAGAAAGA AGAAACCCAG
P. macrorhiza B funct    CATACCACAA AAGAAAAAAG AAAACAAGAA GAAAGAGAAA GAAGAAACCC AGAAAAAAGA GAAAGAAGAA AACAAGAAGA AAGAGAAAGA AGACACCCAG
```

Additional File 1C - *matR* alignment

```
                                    410        420        430        440        450        460        470        480        490        500
                          ....|....| ....|....| ....|....| ....|....| ....|....| ....|....| ....|....| ....|....| ....|....| ....|....|
Nicotiana                 ---------- ---------- ---------- ---------- ---------- ---------- ---------- ---------- ---------- ----------
Convolvulus               ---------- ---------- ---------- ---------- ---------- ---------- ---------- ---------- ---------- ----------
Dinetus                   ---------- ---------- ---------- ---------- ---------- ---------- ---------- ---------- ---------- ----------
C. europaea               ---------- ---------- ---------- ---------- ---------- ---------- ---------- ---------- ---------- ----------
P. coronopus C pseudo     ---------- ---------- ---------- ---------- ---------- ---------- ---------- ---------- ---------- ----------
P. coronopus D pseudo     ---------- ---------- ---------- ---------- ---------- ---------- ---------- ---------- ---------- ----------
P. macrorhiza A pseudo    ---------- ---------- ---------- ---------- ---------- ---------- ---------- ---------- ---------- ----------
P. macrorhiza B pseudo    ---------- ---------- ---------- ---------- ---------- ---------- ---------- ---------- ---------- ----------
P. subspathulata pseudo   ---------- ---------- ---------- ---------- ---------- ---------- ---------- ---------- ---------- ----------
Jovellana                 ---------- ---------- ---------- ---------- ---------- ---------- ---------- ---------- ---------- ----------
Streptocarpus             ---------- ---------- ---------- ---------- ---------- ---------- ---------- ---------- ---------- ----------
Mimulus                   ---------- ---------- ---------- ---------- ---------- ---------- ---------- ---------- ---------- ----------
Aragoa                    ---------- ---------- ---------- ---------- ---------- ---------- ---------- ---------- ---------- ----------
Digitalis                 ---------- ---------- ---------- ---------- ---------- ---------- ---------- ---------- ---------- ----------
Globularia                ---------- ---------- ---------- ---------- ---------- ---------- ---------- ---------- ---------- ----------
P. lanceolata             ---------- ---------- ---------- ---------- ---------- ---------- ---------- ---------- ---------- ----------
P. crassifolia            AGAAAGAAGG AGGAGACAGA AGAAAGGAAA AGGAAGAATC AGAGAAGAGA GGACAGAGAA GAGAAAGAGA GAGAATAGGA ATAGAGAGAA GAGAAAGCAA
P. coronopus C funct      AA-------- ---------- ---------- ---------- ---------- ---------- -----GAAGA GAAGACAGGA ---------- ----------
P. macrorhiza B funct     AA-------- ---------- ---------- ---------- ---------- ---------- -----GAAGA GAAGACAGGA ---------- ----------

                                    510        520        530        540        550        560        570        580        590        600
                          ....|....| ....|....| ....|....| ....|....| ....|....| ....|....| ....|....| ....|....| ....|....| ....|....|
Nicotiana                 ---------- ---------- ---------- ---------- ---------- ---------- ---------- ---------- ---------- ----------
Convolvulus               ---------- ---------- ---------- ---------- ---------- ---------- ---------- ---------- ---------- ----------
Dinetus                   ---------- ---------- ---------- ---------- ---------- ---------- ---------- ---------- ---------- ----------
C. europaea               ---------- ---------- ---------- ---------- ---------- ---------- ---------- ---------- ---------- ----------
P. coronopus C pseudo     ---------- ---------- ---------- ---------- ---------- ---------- ---------- ---------- ---------- ----------
P. coronopus D pseudo     ---------- ---------- ---------- ---------- ---------- ---------- ---------- ---------- ---------- ----------
P. macrorhiza A pseudo    ---------- ---------- ---------- ---------- ---------- ---------- ---------- ---------- ---------- ----------
P. macrorhiza B pseudo    ---------- ---------- ---------- ---------- ---------- ---------- ---------- ---------- ---------- ----------
P. subspathulata pseudo   ---------- ---------- ---------- ---------- ---------- ---------- ---------- ---------- ---------- ----------
Jovellana                 ---------- ---------- ---------- ---------- ---------- ---------- ---------- ---------- ---------- ----------
Streptocarpus             ---------- ---------- ---------- ---------- ---------- ---------- ---------- ---------- ---------- ----------
Mimulus                   ---------- ---------- ---------- ---------- ---------- ---------- ---------- ---------- ---------- ----------
Aragoa                    ---------- ---------- ---------- ---------- ---------- ---------- ---------- ---------- ---------- ----------
Digitalis                 ---------- ---------- ---------- ---------- ---------- ---------- ---------- ---------- ---------- ----------
Globularia                ---------- ---------- ---------- ---------- ---------- ---------- ---------- ---------- ---------- ----------
P. lanceolata             ---------- ---------- ---------- ---------- ---------- ---------- ---------- ---------- ---------- ----------
P. crassifolia            AACAAAAACC AAAACAAAAA GACAGAGGAC AAAAGAAAGA AAAAGAACAT GCGAGACAGA CAGAGAGACA GAGAGACAGA GAGAGAGATC TAGTCCCGGA
P. coronopus C funct      -GAGAAAAGA CAAACAGAAA GACAGAGAAA AACAAAGAGA AAGAAA---- ---------- ----GAGACA GAGAGACAG- ---------- -------GGA
P. macrorhiza B funct     -GAGAAAAGA CAAACAGAAA GACAGAGAAA AACAAAGAGA AAGAAA---- ---------- ----GAGACA GAGAGACAG- ---------- -------GGA

                                    610        620        630        640        650        660        670        680        690        700
                          ....|....| ....|....| ....|....| ....|....| ....|....| ....|....| ....|....| ....|....| ....|....| ....|....|
Nicotiana                 ---------T CGTTGTTGCA TAGCTCAGGT CGGAGCAAGG TGCCATCGGA CGTTCAA--- ---CAGGTAG TCTCACGATC GGGCACTCAT GCCCCGACAT
Convolvulus               ---------. .......... .......... .......... .......... .......--- ---....C.. .......... .....TGAGG ....G..A..
Dinetus                   ---------. .......... .......... .......... .......... .......--- ---....C.. .......... .....TGAGG ....G..A..
C. europaea               ---------. .......... .......... .......... .......... .......--- ---....C.. .......... .....TGAGG ....G..A..
P. coronopus C pseudo     ---------. ......C.T. ..A....... .......TA. ....G...A. .......--- ---....C.. ...T...... .....TAAGG AT..G..A..
P. coronopus D pseudo     ---------. .......... .......... .......... ..T...A... ...C...--- ---....C.. .......... ......GAGG ....G..A..
P. macrorhiza A pseudo    ---------. .......A.. .......... .......... ..T...A... ...C...CAA Axx....C.. .......... .....TGAGG ....G..A..
P. macrorhiza B pseudo    ---------. .......... .......... .......... ......A... T..C.G.--- ---....C.. .......... .....TAAGG ....G..A..
P. subspathulata pseudo   ---------- ---------- ---------- ---------- ---------- ---------- ---------- ---------- ---------- ----------
Jovellana                 ---------. .......... .......... .......... .......... .......--- ---....C.. .......... .......... ....G..AG.
Streptocarpus             ---------. .......... .......... .......... .......... .......--- ---....C.. .......... ..T....... ....G..AG.
Mimulus                   ---------. .......... .......... .......... .......... .......--- ---....C.. .......... .......... ....G..AG.
Aragoa                    ---------. .......... .......... .TCC...... .......... .......--- ---....C.. .......... .......... ....G..AG.
Digitalis                 ---------. .......... .......... .TCC...... .......... .......--- ---....C.. .......... .......... ....G..AG.
Globularia                ---------. .......... .......... .TCC...... .......... .......--- ---....C.. .......... .......... ....G..AG.
P. lanceolata             ---------. TC........ .......... TTCC....A. GC..T..... .......--- ---G..---- -----..... .A........ .......AGG
P. crassifolia            AAAAGCCAA- ---------- ---------- ---------- ---------- ---------- ---------- ---------- ---------- ----------
P. coronopus C funct      AAA------- ---------- ---------- ---------- ---------- ---------- ---------- ---------- ---------- ----------
P. macrorhiza B funct     AAA------- ---------- ---------- ---------- ---------- ---------- ---------- ---------- ---------- ----------

                                    710        720        730        740        750        760        770        780        790        800
                          ....|....| ....|....| ....|....| ....|....| ....|....| ....|....| ....|....| ....|....| ....|....| ....|....|
Nicotiana                 TGTCATTGTA TACTCCCGCG GGTCGGAAGG CGGCGGGGGA AGGAGGGGGA CACTGGGCGA GATCTATCAG CAGCGAATTC CCCATACAAA TAGAGGCACC
Convolvulus               .TG....... .......... .......... .......... .......... .......... .......... .......... .......... .......G..
Dinetus                   .TG....... .......... .......... .......... .......... .......... .......... .......... .......... .......G..
C. europaea               .T........ .......... .......... .........G .......... .......... .......... .......... .......... ..........
P. coronopus C pseudo     .TG....A.. .......... .......... .....----- --------.. .......T.. .......... .......... .........T AG........
P. coronopus D pseudo     .TG....... ......TAA. ........A. ........C. ....AA...G ......AT.. .......T.. T......... T........T .........T
P. macrorhiza A pseudo    .TG....... .......AA. .......... .A......T. ........AG ....AA.... .......TT. T...A..... .........T .....-----
P. macrorhiza B pseudo    .TG....... .......... .......... ...T....TC .....AA... ......A... .......T.. T......... .........T ..........
P. subspathulata pseudo   ---------- ---------- .......... ........C. .........G ......AT.. .......T.. T......... T........T .........T
Jovellana                 .......... .......... .......... .......... .......... .......... .......... .......... .......... .G.....G..
Streptocarpus             .......... .......... .......... .......... .......... .......... .......... .......... .......... .G.G...G..
Mimulus                   .......... .......... .......... .......... .......... .......... .......... .........G .......... .G.....G..
Aragoa                    .......... .......... .......... .........G .......... .......... .......... ...A...... .......... .G.....G..
Digitalis                 .......... .......... ...T...... .......... .......... .......... .......... ...A...... .......... .G.....G..
Globularia                .......... .......... .......... .......... .......... .......... .......... ...A...... .......... .G.....G..
P. lanceolata             .......... ......AA.A ...A...G.. .TT.A..... .A.G.....G .......... ........CA ...AA....T ..T....... .......G..
P. crassifolia            ---------- ---------- ---------- ---------- ---------- ---------- ---------- ---------- ---.AC.... ..........
P. coronopus C funct      ---------- ---------- ---------- ---------- ---------- ---------- ---------- ---------- ---...AG.. ....A.....
P. macrorhiza B funct     ---------- ---------- ---------- ---------- ---------- ---------- ---------- ---------- ---...AG.. ....A.....
```

Additional File 1C - *matR* alignment

```
                                810        820        830        840        850        860        870        880        890        900
                         ....|....| ....|....| ....|....| ....|....| ....|....| ....|....| ....|....| ....|....| ....|....| ....|....|
Nicotiana                TATCAAAAAG ATACTTCGAA GGCTTCGGGA TCGAGGTCTC ATTAGCCGAA GAAGACCCTG GCCAATCCAC GTGGCCTGCT TGACGAACGT CAGCGACGGA
Convolvulus              .......... .....C.... .......... .......... .......... .......... .......... .......... .......... ...T......
Dinetus                  .......... .....C.... .......... .......... .......... .......... .......... .......... .......... ...T......
C. europaea              .......... .....C.... .......... .......... .......... .......... .......... .......... .......... A..T......
P. coronopus C pseudo    .......... .....C.A.. A...CT...- ---------. .......... .......... A......T.. ...AT.C.T. ....A..... A..T------
P. coronopus D pseudo    .........T .....C...G A.....AA.. ...C...... .........G .......... .......... .......... ......G... A..TA.....
P. macrorhiza A pseudo   ----.....T .....C.... A......... .......... .........G .......... .......... .......... ......G... A..TA.....
P. macrorhiza B pseudo   .........T ...T.C.... A......... .....A.... ....C..... .......... .........T ...A...... .C........ A..TA.....
P. subspathulata pseudo  .........T .....C...G A.....AA.. ...C...... .........G .......... .......... .......... ......G... A..TA.....
Jovellana                .......... .....C.... .......A.. .......... .......... .......... .......... .......... .......... ..........
Streptocarpus            .......... .....C.... .......... .......... .......... .......... .......... .......... .......... ..........
Mimulus                  .......... .....C.... .......... .......... .......... .......... .......... .......... .......... ........A.
Aragoa                   .......... .....C.... .......... .......... .......... .......... .......... .......... .......... ..........
Digitalis                .......... .....C.... .......... .......... .......... .......... .......... .......... .......... ..........
Globularia               .......... .....C.... .......... .......... .......... .......... .......... .......... .......... ..........
P. lanceolata            ...A...... .....C.... A......... .......... .......... .......... ...T...... .......... .A....T... ..........
P. crassifolia           C......... ...G.CA... ....CA.A.. CA....GA.. .......C.. .......A.. ...T.....T TGC....C.. ....C...CA A......C..
P. coronopus C funct     C..A...... ..T..C.A.. ....CA.ACT CAT...AA.. ......AA.. A......T.. ...C..A..T .CAC..CAA. ....A...CA A.C....AC.
P. macrorhiza B funct    C..A...... ..T..C.A.. ....CA.ACT CAT...AA.. ......AA.. A......T.. ...C..A..T .CAC..CAA. ....A...CA A.C....AC.

                                910        920        930        940        950        960        970        980        990       1000
                         ....|....| ....|....| ....|....| ....|....| ....|....| ....|....| ....|....| ....|....| ....|....| ....|....|
Nicotiana                GACATCGTAA ATTGGTCCGC GGGCATCGCG ATAAGTCCTC TGTCCTACTA CAGGTGCTGC GACAACCTTT ACCAAGTCCG AACGATTGTC GACCACCAGA
Convolvulus              .......... .......... .......... .......... .......... .......C.. .......... .......... .......... ...T......
Dinetus                  .......... .......... .......... .......... .......... .......C.. .......... .......... .......... ...T......
C. europaea              .......... .......... .......... .......... .......... .......C.. .......... .......... .......... ...T......
P. coronopus C pseudo    -.....A... ...A...... .A........ .G.......T .......... .......C.T .......... .......A.. ....G..... ...T......
P. coronopus D pseudo    .......... .........A ..A....... .C....T..A ..C....... .......C.. .G........ .......... .......... A..T......
P. macrorhiza A pseudo   .......... .......... .......... .C........ ..C....... ......TC.. .......... .......... .......... ...T......
P. macrorhiza B pseudo   ......A... T......T.. ........T. .......... .......... ......TCA. .......... .......... ....C..... ...T.T....
P. subspathulata pseudo  .......... .......... ..A....... .C.......A ..C....... .......C.. .......... .......... .......... A..T......
Jovellana                .......... .......... .......... ..T....... .......... .......C.. .......... .......... ...A...... A.........
Streptocarpus            .......... .......... .......... .......... .......... .......C.T .......... .......... ...A...... ..........
Mimulus                  .......... .......... .......... .......... .......... .......CA. .......... .......... ...A...... ..........
Aragoa                   .......... .......... ......T... .......... .......... .......C.. .......... .......... ...A...... ..........
Digitalis                .......... .......... .......... .......... .......... .......C.. .......... .......... ...A...... ..........
Globularia               .......... .......... .......... .......... .......... .......C.. .......... .......... ...A...... ..........
P. lanceolata            .......... ......A... ......T... .......T.T ...T...... T.A..A.C.. ........G. .......... ..GA...... ...T......
P. crassifolia           ........CC .G....A... ......T... .G......
P. coronopus C funct     ........CG .A....A... AC....T..C ....AAA.CT ...A...... ..AAGAAC.. A.A...TGCA CA......AA ..GA..C..
P. macrorhiza B funct    ........CG .A....A... AC....T..C ....AAA.CT ...A...... ..AAGAAC.. A.A...TGCA CA......AA ..GA..C..

                               1010       1020       1030       1040       1050       1060       1070       1080       1090       1100
                         ....|....| ....|....| ....|....| ....|....| ....|....| ....|....| ....|....| ....|....| ....|....| ....|....|
Nicotiana                TCCGCTGGTC TGCAATATTC ACCCCGGCCC ACAAGCACAA ATCCTCGGCG CGGAATATAA TCCTAAAGTA CTCCAAAGAC TCAAATATAG TCAATCAAGA
Convolvulus              .......... .......... .......... .......... .......... ..A....... .......... .......... ...C...... ..........
Dinetus                  .......... .......... .......... .......... .......... ..A....... .......... .......... ...C...... ..........
C. europaea              .......... .......... .......... .......... .......... ..A....... .......... .......... ...C...... ..........
P. coronopus C pseudo    ...A...... .......... G......... .......... .......... ..A....... .......... T......... ..CT...... .....A....
P. coronopus D pseudo    ...TT....T .......... .T........ .....T.... ........T. T.A....... .T.....A.. .......... ...C...... .....A....
P. macrorhiza A pseudo   ....T....T .......... .......... .......... ........T. T.A....... .T........ .......... .......... .....A....
P. macrorhiza B pseudo   ..T....... .......... .......... .......... .......... .......... .......... .......... ..GC...... .....A....
P. subspathulata pseudo  ....T.---- ---------- ---------- ---------- ---------- ---------- ---------- ---------- ---------- -----A....
Jovellana                .......... .......... .......... .......... .......... .......... ...C...... .......... .......... ..........
Streptocarpus            .......... .........A .......... .......... ..G....... .......... ...C...... .......... .......... ........A.
Mimulus                  .......... .......... .......... .......... .......... .......... ...C...A.. .......... .......G.. ..........
Aragoa                   .......... .......... .......... .......... .......... .......... ...C...... .......... .......... ..........
Digitalis                .......... .......... .......... .......... .......... .......... ...C...... .......... .......... ..........
Globularia               .......... .......... .......... .......... .......... .......... ...C...... .......... .......... ..........
P. lanceolata            .......... C......G.T ...T....T. .T..A..... .A......T. .......... ...C...... .......... .......... ..........
P. crassifolia
P. coronopus C funct
P. macrorhiza B funct

                               1110       1120
                         ....|....| ....|....| ....|...
Nicotiana                AGGTGGTAAG ACCCTTGCAG AGTTCCCC
Convolvulus              .......... .......... .....T..
Dinetus                  .......... .......... .....T..
C. europaea              .......... .......... .....T..
P. coronopus C pseudo    ----...... ...T....G. .....T..
P. coronopus D pseudo    ----...... .......... .....T..
P. macrorhiza A pseudo   ----...... .......... .....T..
P. macrorhiza B pseudo   ----.A.... .......... .....T..
P. subspathulata pseudo  ----...... .......... .....T..
Jovellana                ...C...... .......... .A......
Streptocarpus            .......... .......... .A......
Mimulus                  ..AC...... .T......G. .A......
Aragoa                   ...C...... .......... .A......
Digitalis                ...C...... .......... .A......
Globularia               ...C...... .......... .A......
P. lanceolata            .A........ ......T... .A..T...
P. crassifolia
P. coronopus C funct
P. macrorhiza B funct
```
