# Supplementary material for: Horizontal acquisition of multiple mitochondrial genes from a parasitic plant followed by gene conversion with host mitochondrial genes
Source: BMC Biol. 2010 Dec 22;8:150. doi: 10.1186/1741-7007-8-150 (PMC3022774; doi:10.1186/1741-7007-8-150)
Supplement: Additional file 2 — GenBank accession numbers. Accessions are provided for all newly generated as well as previously available sequences used in this study. [file 1741-7007-8-150-S2.PDF]

Additional File 2A - GenBank accession numbers for Plantaginaceae sequences

	foreign copies			native copies					
**Species**	***atp1***	***atp6***	***matR***	***atp1***	***atp6***	***matR***	***rbcL***	***trnL/trnF***	**ITS**
P. coronopus A	HQ593797	HQ593764	HQ593736	-	-	-	HQ593824	HQ593815	HQ593830
P. coronopus B	AY741850	HQ593765	HQ593737	AY741843	-	-	HQ593825	HQ593816	HQ593831
P. coronopus C	HQ593798	HQ593766	HQ593738	HQ593802	HQ593770	HQ593750	HQ593826	HQ593817	HQ593832
P. coronopus D	HQ593799	HQ593767	HQ593739	HQ593803	-	HQ593751	HQ593827	HQ593818	HQ593833
P. macrorhiza A	AY741852	HQ593768	HQ593740	AY741845	-	-	-	HQ593819	HQ593835
P. macrorhiza B	HQ593800	HQ593769	HQ593741	HQ593804	HQ593771	HQ593752	HQ593828	HQ593820	HQ593834
P. subspathulata	AY741851	-	HQ593742	AY741846	-	-	-	HQ593821	HQ593836
P. crassifolia	-	-	-	AY741844	HQ593772	HQ593749	-	AY101936	AY101881
P. lanceolata	-	-	-	AY818937	HQ593773	HQ593747	L36454	AY101952	AY101898
P. maritima	-	-	-	HQ593805	HQ593774	HQ593748	HQ593829	HQ593822	HQ593837
P. sericea	-	-	-	AY818941	-	-	-	-	-
Aragoa abietina	-	-	-	HQ593801	HQ593775	HQ593743	AJ459242	HQ593823	AJ459404
Digitalis purpurea	-	-	-	AY741841	HQ593776	HQ593744	L01902	AY591291	AY591257
Globularia salicina	-	-	-	AY741842	HQ593778	HQ593745	-	-	-
Veronica incana	-	-	-	AY818943	HQ593777	HQ593746	-	-	-

Additional File 2B - GenBank accession numbers for non-Plantaginaceae sequences

atp1		***atp6***		***matR***	
**Species**	**Acc. No.**	**Species**	**Acc. No.**	**Species**	**Acc. No.**
Ajuga reptans	AY818931	*Ajuga reptans*	HQ593780	*Asclepias tuberosa*	AY453103
Asclepias viridis	EU280981	*Catalpa bignonioides*	HQ593779	*Blepharis hildebrandtii*	AF520153
Catalpa bignonioides	AY741840	*Ceropegia woodii*	HQ593793	*Clerodendrum inerme*	AY289669
Coffea arabica	DQ401313	*Clerodendrum sp*	HQ593781	*Coffea arabica*	DQ401399
Convolvulus assyricus	AY596678	*Convolvulus assyricus*	HQ593790	*Convolvulus assyricus*	HQ593758
Cuscuta campestris	AY940733	*Cuscuta europaea*	HQ593788	*Cuscuta europaea*	HQ593759
Cuscuta europaea	AY596701	*Cuscuta exaltata*	HQ593789	*Cuscuta gronovii*	HQ593761
Cuscuta japonica	AY596702	*Cuscuta gronovii*	HQ593786	*Cuscuta japonica*	EU281124
Cuscuta sandwichiana	AY741820	*Cuscuta sandwichiana*	HQ593787	*Cuscuta sandwichiana*	HQ593760
Dinetus truncatus	AY596699	*Dinetus truncatus*	HQ593791	*Dinetus racemosa*	HQ593757
Frasera caroliniensis	EU280966	*Hoya carnosa*	HQ593794	*Frasera caroliniensis*	EU281106
Humbertia madagascariensis	AY741819	*Jovellana sp*	HQ593783	*Ipomoea alba*	EU281111
Ipomoea batatas	AY596672	*Mimulus guttatus*	HQ593782	*Jacaranda mimosifolia*	AF520145
Jasminum floridum	EU280978	*Montinia caryophyllacea*	HQ593792	*Jasminum abyssinicum*	AF520152
Mimulus guttatus	EU551652	*Nerium oleander*	HQ593795	*Jovellana sp*	HQ593754
Montinia caryophyllacea	AY596706	*Nicotiana tabacum*	BA000042	*Mimulus guttatus*	HQ593753
Myoporum sandwicense	AY741838	*Olea europaea*	HQ593785	*Montinia caryophyllacea*	HQ593762
Nicotiana tabacum	BA000042	*Pentas lanceolata*	HQ593796	*Nicotiana tabacum*	BA000042
Orobanche fasciculata	AY741831	*Petunia axillaris*	S75449	*Orobanche uniflora*	DQ110351
Paulownia tomentosa	AY741826	*Solanum tuberosum*	AF095276	*Schizanthus pinnatus*	HQ593763
Petunia axillaris	U61392	*Streptocarpus holstii*	HQ593784	*Solanum tuberosum*	AJ003130
Schizanthus pinnatus	AY596705			*Streptocarpus holstii*	HQ593755
Sesamum indicum	AY741827			*Syringa vulgaris*	HQ593756
Streptocarpus holstii	AY741823			*Vinca minor*	AF520143
Strobilanthes dyeriana	AY741825				
Strychnos spinosa	AY741818				
Syringa vulgaris	AY741821				

Additional File 2C - GenBank accession numbers for cDNA sequences

atp1		*atp6*		*matR*	
**Species**	**Acc. No.**	**Species**	**Acc. No.**	**Species**	**Acc. No.**
Arabidopsis thaliana	Y08501	*Arabidopsis thaliana*	Y08501	*Arabidopsis thaliana*	Y08501
Beta vulgaris	BA000009	*Beta vulgaris*	BA000009	*Beta vulgaris*	BA000009
Citrullus lanatus	GQ856147	*Citrullus lanatus*	GQ856147	*Citrullus lanatus*	GQ856147
Oenothera biennis	X04023	*Oenothera berteriana*	Y00465	*Oenothera berteriana*	M63034
Oryza sativa	BA000029	*Oryza sativa*	BA000029	*Vitis vinifera*	ref [52]
Vitis vinifera	ref [52]	*Vitis vinifera*	ref [52]	*Zea mays*	AY506529
