# Supplementary material for: Horizontal acquisition of multiple mitochondrial genes from a parasitic plant followed by gene conversion with host mitochondrial genes
Source: BMC Biol. 2010 Dec 22;8:150. doi: 10.1186/1741-7007-8-150 (PMC3022774; doi:10.1186/1741-7007-8-150)
Supplement: Additional file 3 — Sources of material. Sources, type of material, and voucher information for Plantago species used in this study. [file 1741-7007-8-150-S3.PDF]

Additional File 3 - Sources of *Plantago* material

Species Line	Material	Source	Source ID	Voucher
P. coronopus A	DNA	Royal Botanic Gardens, Kew (UK)	Kew 2763	Chase 2763 K
P. coronopus B	DNA	Royal Botanic Gardens, Kew (UK)	Kew 9439	Ronsted 8 C
P. coronopus C	seed	Western Regional Plant Introduction Station (USA)	W6 4759	Mower 500 NEB
P. coronopus D	seed	B&T World Seeds (France)	15416	Mower 505 NEB
P. crassifolia	DNA	Royal Botanic Gardens, Kew (UK)	Kew 9393	Ronsted 17 C
P. lanceolata	DNA	Royal Botanic Gardens, Kew (UK)	Kew 2765	Chase 2765 K
P. macrorhiza A	DNA	Royal Botanic Gardens, Kew (UK)	Kew 9614	Chase 9614 K
P. macrorhiza B	seed	National Botanical Garden (Belgium)	20051259-01	Mower 511 NEB
P. maritima	seed	Western Regional Plant Introduction Station (USA)	PI 415825	Mower 507 NEB
P. sericea	DNA	Royal Botanic Gardens, Kew (UK)	Kew 2768	Chase 2768 K
P. subspathulata	DNA	Royal Botanic Gardens, Kew (UK)	Kew 9431	none
