# Supplementary material for: Horizontal acquisition of multiple mitochondrial genes from a parasitic plant followed by gene conversion with host mitochondrial genes
Source: BMC Biol. 2010 Dec 22;8:150. doi: 10.1186/1741-7007-8-150 (PMC3022774; doi:10.1186/1741-7007-8-150)

Additional File 4 – Additional phylogenetic analyses.

The *atp1*, *atp6*, and *matR* data sets were re-evaluated in several ways. (A) Neighbor joining was performed using MEGA version 4.0.2 [85] on the original data sets. The analysis used the maximum composite likelihood model, pairwise deletion of gaps, gamma-distributed rate variation among sites, and 500 replicates of bootstrapping. (B) Parsimony was also performed using MEGA on the original data sets. For this analysis, gaps and missing data were not excluded from the data sets, tree space was searched using 10 replicates of random starting trees and the close-neighbor interchange (level=1) heuristic search, and 500 replicates of bootstrapping were performed. (C) Edit sites and codons affected by gene conversion were eliminated from the data sets, and then maximum likelihood was performed as described in the main text. (D) The native functional copies from all *Plantago* species were removed from the original data sets and then evaluated by maximum likelihood. (E) Additional representatives from asterids, caryophyllids, and rosids were added to the original data sets and then evaluated by maximum likelihood.

Additional File 4A
Neighbor Joining

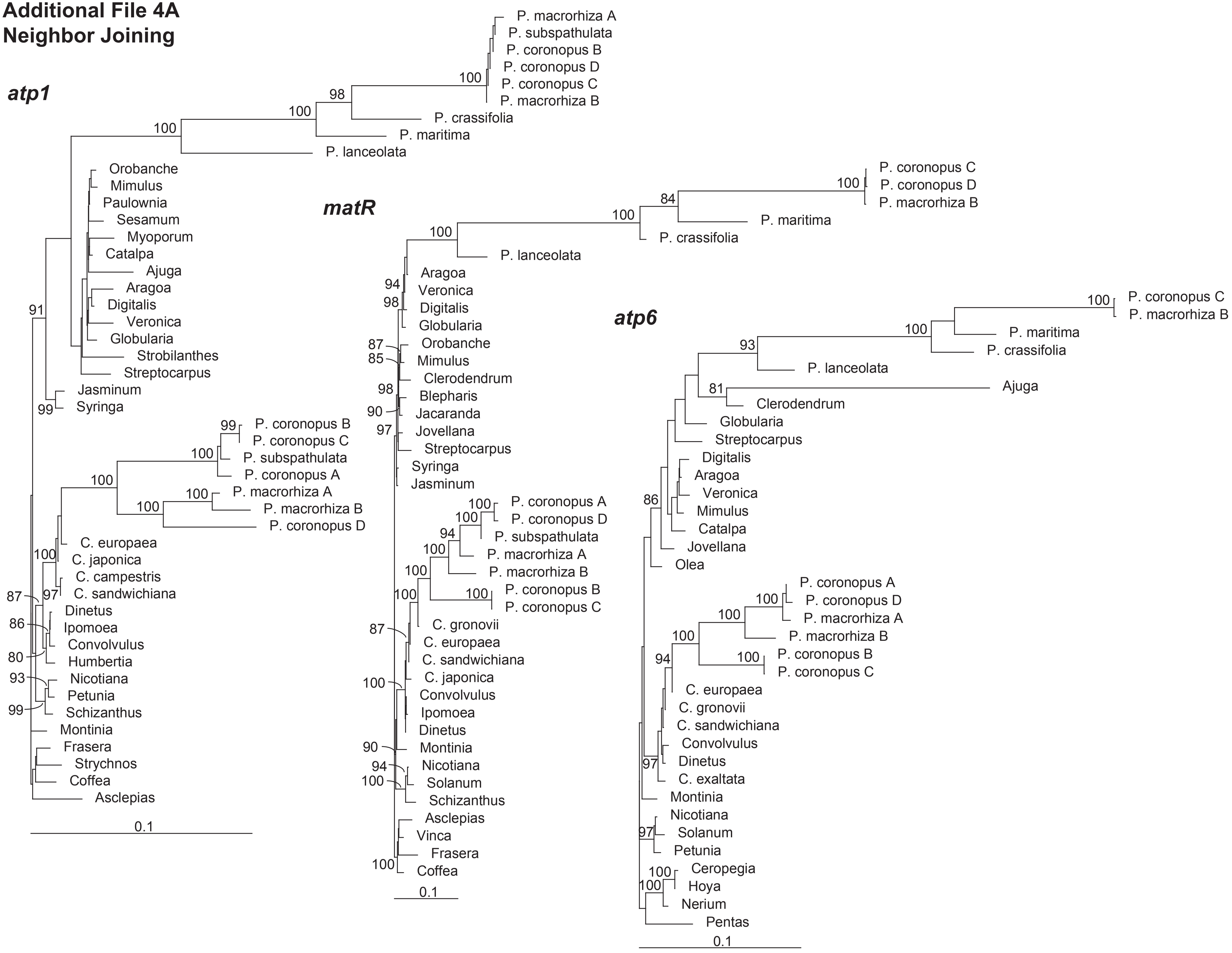

Additional File 4B
Parsimony

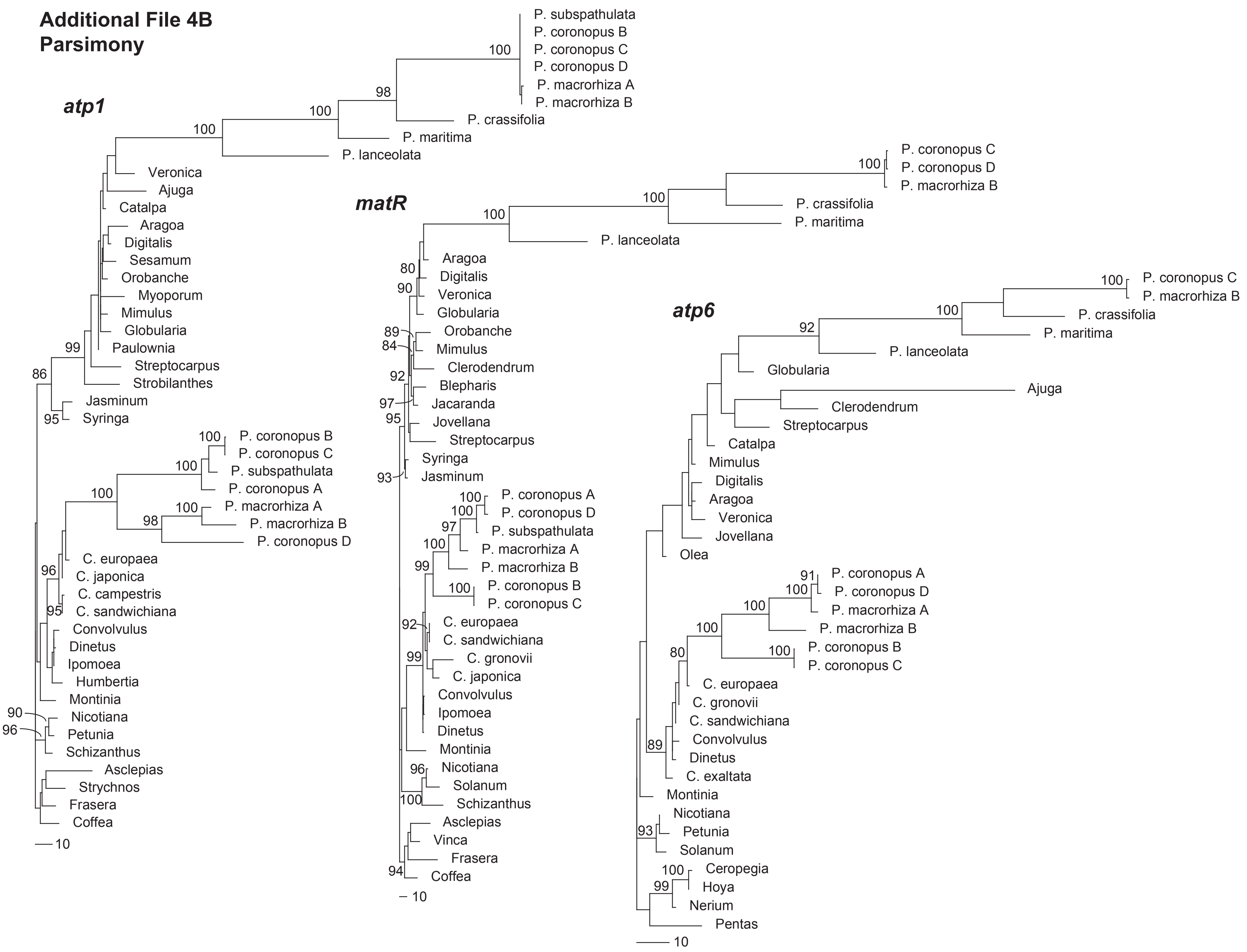

Additional File 4C
Edit Sites and Converted Regions Excluded

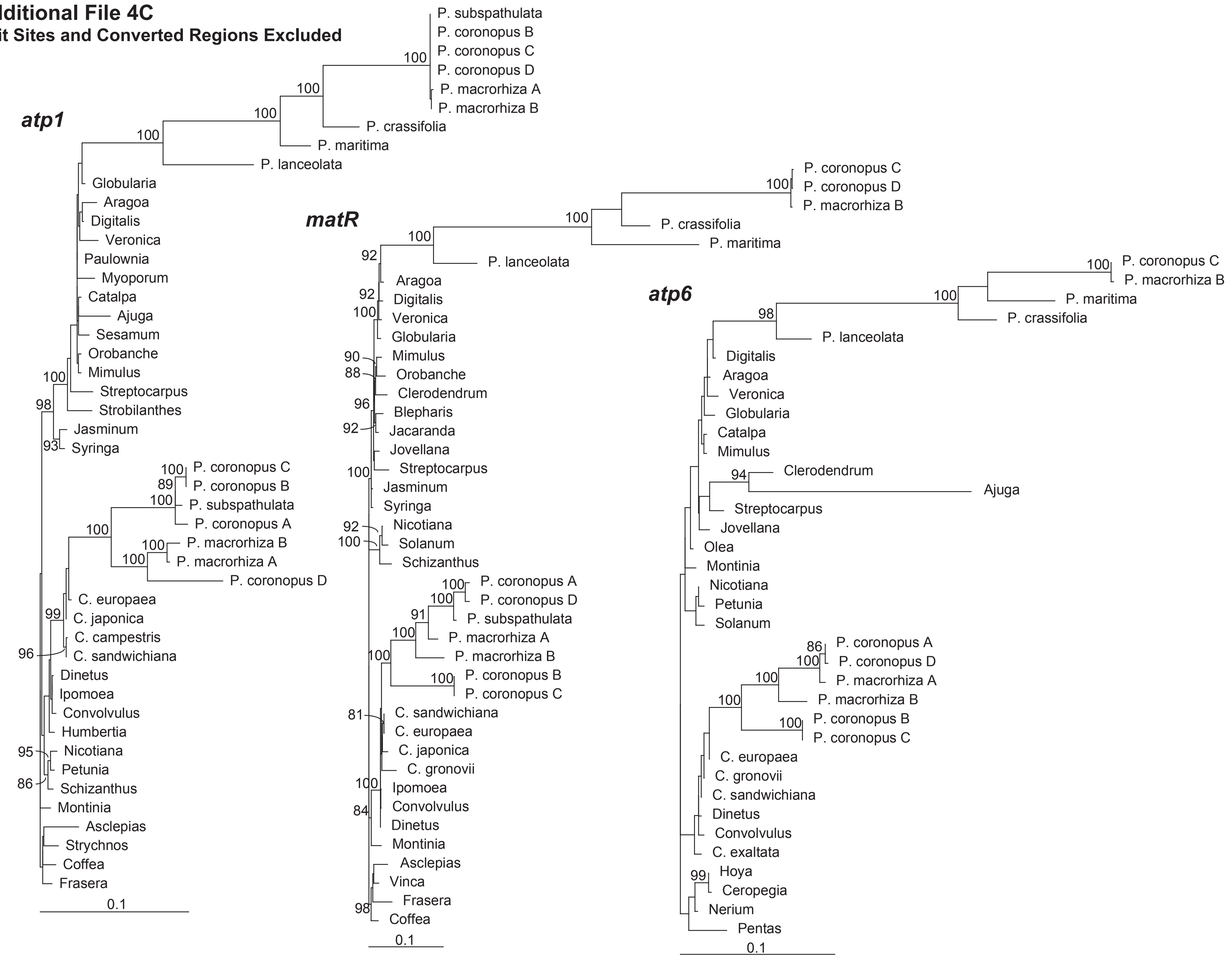

Additional File 4D
Plantago Native Copies Excluded

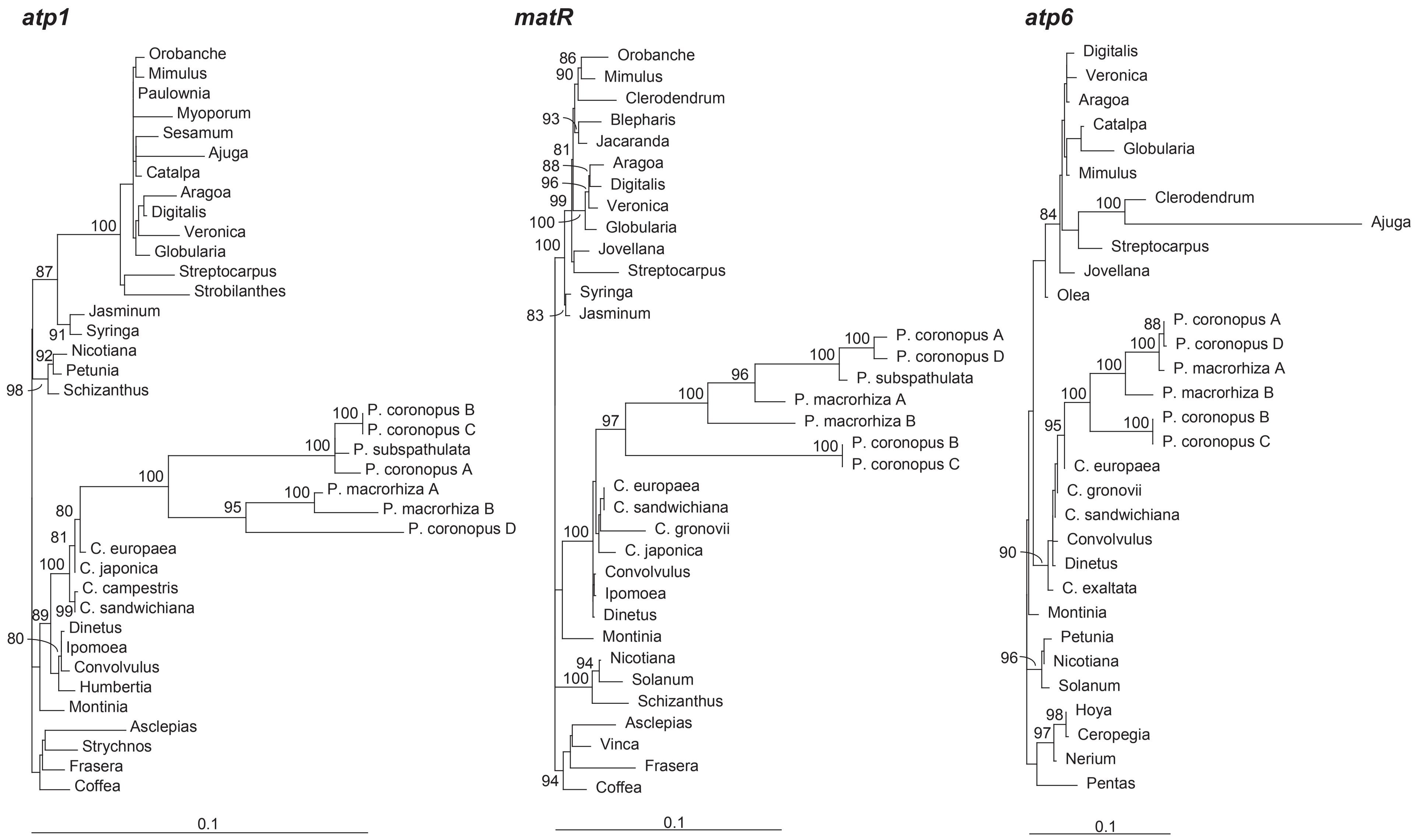

Additional File 4E
Expanded Taxon Set

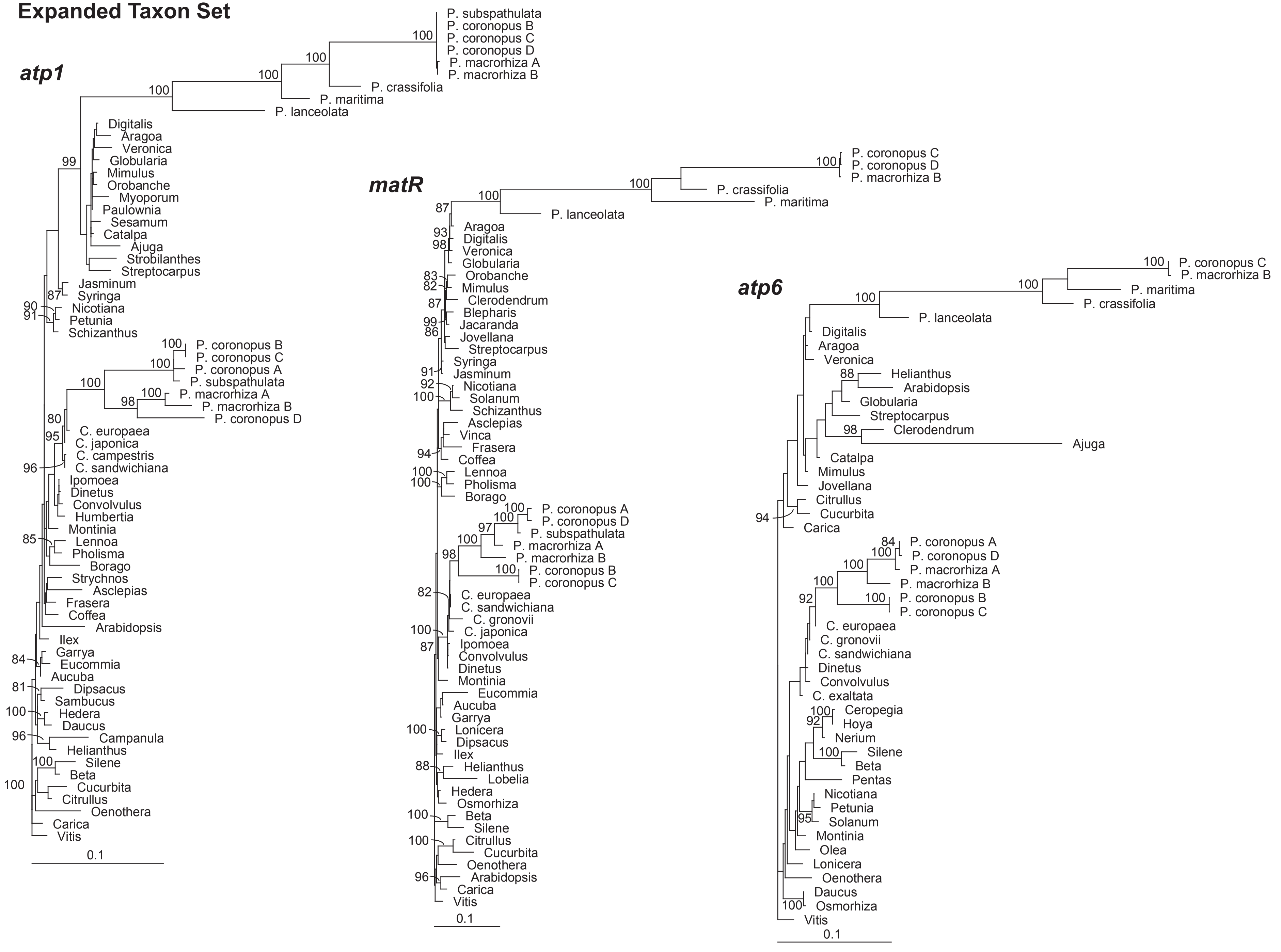

Supplement: Additional file 4 — Additional phylogenetic analyses. The atp1, atp6 and matR data sets were re-evaluated in several alternative ways. [file 1741-7007-8-150-S4.PDF]
