# Supplementary material for: Horizontal acquisition of multiple mitochondrial genes from a parasitic plant followed by gene conversion with host mitochondrial genes
Source: BMC Biol. 2010 Dec 22;8:150. doi: 10.1186/1741-7007-8-150 (PMC3022774; doi:10.1186/1741-7007-8-150)
Supplement: Additional file 5 — Tests of alternative tree topologies. Results of Shimodaira-Hasegawa Tests for comparisons of alternative phylogenetic hypotheses. [file 1741-7007-8-150-S5.PDF]

Additional File 5 – Shimodaira-Hasegawa Test.

The SH Test was run using baseml from PAML version 4.1 on a reduced set of taxa common to all genes. The GTR nucleotide model was used with a gamma distribution of rate variation among sites. Edit sites and codons affected by gene conversion were eliminated from the data sets as described in the main text. The test compared (A) the maximum likelihood topology and (B) the alternative topology in which the pseudogenes arose by duplication. The horizontal pseudo-copies are shown in red. Log likelihood scores are given below each topology for each gene.

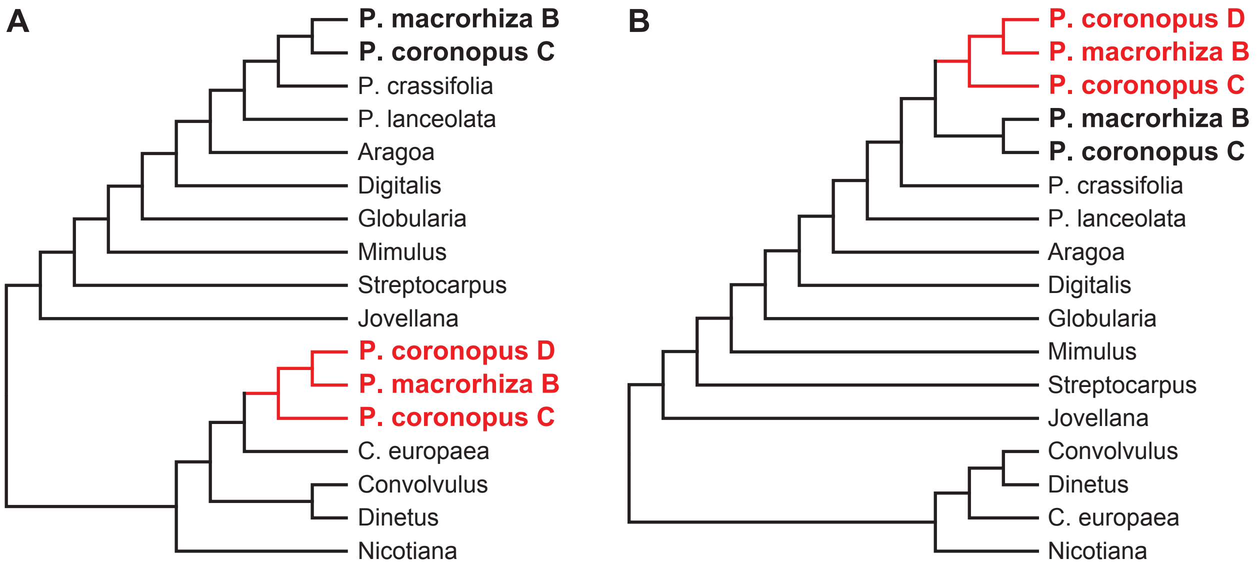

**-lnL =**	**4664.6**	***atp1***	**-lnL =**	**4940.7**
**-lnL =**	**2189.3**	***atp6***	**-lnL =**	**2323.3**
**-lnL =**	**7741.4**	***matR***	**-lnL =**	**8071.1**
